# Beam Search for Automated Design and Scoring of Novel ROR Ligands with Machine Intelligence**

**DOI:** 10.1002/anie.202104405

**Published:** 2021-07-19

**Authors:** Michael Moret, Moritz Helmstädter, Francesca Grisoni, Gisbert Schneider, Daniel Merk

**Affiliations:** ^1^ ETH Zurich Department of Chemistry and Applied Biosciences Vladimir-Prelog-Weg 4 8093 Zurich Switzerland; ^2^ Goethe University Frankfurt Institute of Pharmaceutical Chemistry Max-von-Laue-Strasse 9 60438 Frankfurt Germany; ^3^ Eindhoven University of Technology Institute for Complex Molecular Systems Department of Biomedical Engineering Groene Loper 7 5612AZ Eindhoven Netherlands; ^4^ ETH Singapore SEC Ltd 1 CREATE Way, #06-01 CREATE Tower Singapore 138602 Singapore; ^5^ LMU Munich Department of Pharmacy Butenandtstrasse 7 81377 Munich Germany

**Keywords:** de novo design, deep learning, drug discovery, neural network, nuclear receptor

## Abstract

Chemical language models enable de novo drug design without the requirement for explicit molecular construction rules. While such models have been applied to generate novel compounds with desired bioactivity, the actual prioritization and selection of the most promising computational designs remains challenging. Herein, we leveraged the probabilities learnt by chemical language models with the beam search algorithm as a model‐intrinsic technique for automated molecule design and scoring. Prospective application of this method yielded novel inverse agonists of retinoic acid receptor‐related orphan receptors (RORs). Each design was synthesizable in three reaction steps and presented low‐micromolar to nanomolar potency towards RORγ. This model‐intrinsic sampling technique eliminates the strict need for external compound scoring functions, thereby further extending the applicability of generative artificial intelligence to data‐driven drug discovery.

## Graphical abstract

The beam search algorithm was employed for automated molecule design and scoring from a chemical language model (CLM). Prospective application of this model‐intrinsic technique with a CLM trained on inverse RORγ agonists yielded novel ligands of this nuclear receptor with the intended bioactivity. Beam search sampling overcomes the need for external scoring methods and extends the applicability of machine learning‐driven molecular design.

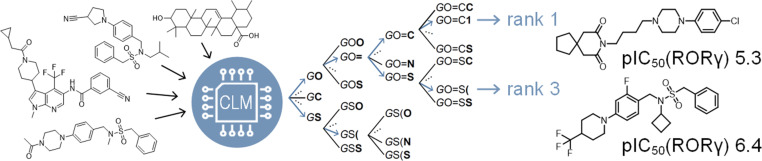

## Introduction

**Figure 1:**
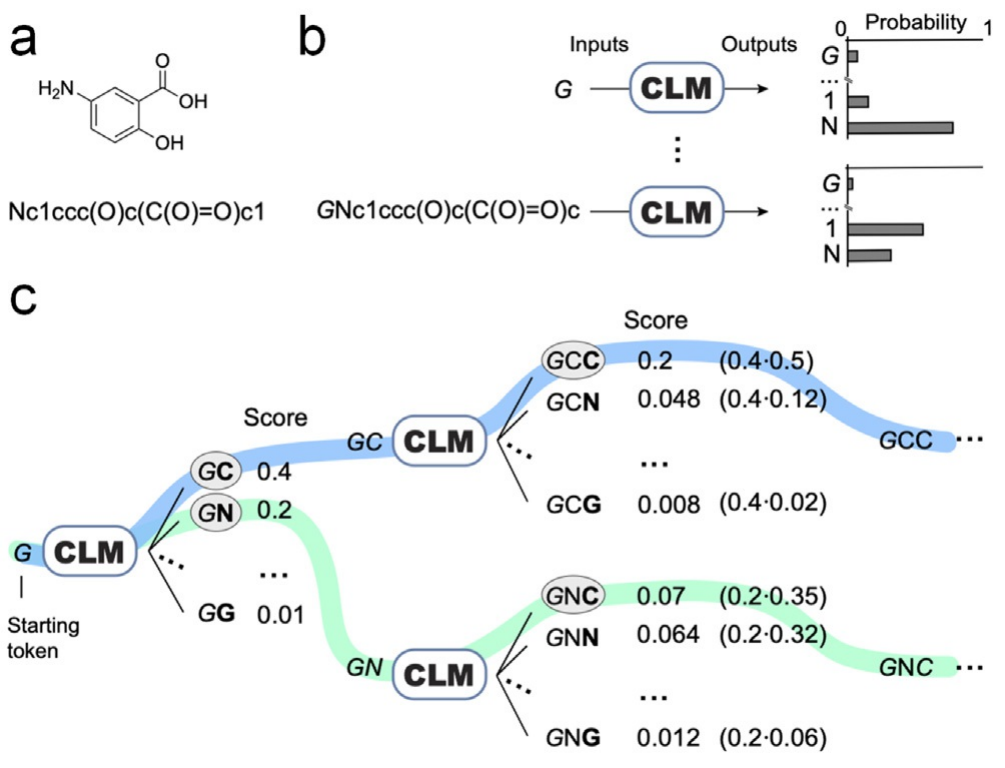
Molecule generation with a chemical language model (CLM) and beam search sampling. a) Kekulé structure of an example molecule and corresponding SMILES string. b) CLM training. The CLM learns to predict the probability of each SMILES string character (“token”) based on the previous tokens in the string. c) Beam search decoding of width two (*k*=2): The design algorithm keeps track of the two most likely SMILES strings (highlighted in color). In this example, the SMILES string generation proceeds from left to right.

Generative deep learning,[^1^, ^2^] that is, a class of machine learning models able to generate new data, can be applied to computationally design pharmacologically active compounds de novo.[^3^, ^4^, ^5^] Deep learning‐based molecular design algorithms can extract high‐level molecular features from “raw” molecular representations,[^6^, ^7^, ^8^, ^9^, ^10^] such as molecular graphs and the Simplified Molecular Input Line Entry System (SMILES, Figure 1 a),^[11]^ potentially allowing them to access unexplored regions of the chemical space.^[12]^ Previous studies showed that chemical language models (CLMs),[^13^, ^14^] in particular generative deep learning models trained on SMILES strings, can generate novel molecules with experimentally validated bioactivity.[^9^, ^15^, ^16^] CLMs have shown the ability to learn focused chemical features from small collections of template molecules by means of transfer learning, that is, a method to reuse previously learned knowledge on a new task for which the available data is scarce.[^15^, ^17^, ^18^] Transfer learning is performed in two steps. In the first step, a model is trained on a large amount of data that relate to the task to be performed (“pre‐training”). In the case of CLMs, this is usually done using large collections of molecules (e.g., in the order of 200 000 to 1 000 000[^9^, ^16^, ^17^]). Pre‐training enables the generative model to capture a) the SMILES “syntax” (i.e., how alphanumeric characters should be assembled to generate strings that correspond to valid molecules, Figure 1) and b) the properties of the pre‐training dataset, such as physicochemical features and synthesizability of the molecules in the dataset. In the second step, the pre‐trained CLM is further trained (“fine‐tuned”) with a smaller set of task‐specific molecules.[^13^, ^19^, ^20^] During this transfer learning process, the CLM is biased towards the chemical space of interest, that is, molecules with desired biological and physicochemical properties. This ability to learn in a low‐data regime (“few‐shot” learning[^21^, ^22^]) renders CLMs particularly useful for application to biological targets for which only few ligands are known. The fully trained CLM can be used to generate new molecules in the form of SMILES strings. Such data generation is performed by predicting one character of a SMILES string (“token”) at a time, based on all the previous tokens. Importantly, this process does not require handcrafted molecule design rules, as CLMs learn solely from the SMILES strings used for training.

Previous prospective applications of CLMs for de novo molecule generation used the so‐called “temperature sampling” to generate large virtual molecular libraries.[^9^, ^13^, ^15^] Temperature sampling allows to sample new SMILES strings by adding tokens to the (growing) string according to the probabilities learned by the CLM, wherein the most likely token at a given position will be sampled more often (Figure 1 b). However, the generated SMILES strings might not always be “chemically meaningful” (invalid strings), or they might not match the feature distribution of the training data because of the random component of temperature sampling. Therefore, additional methods are usually needed to select the most promising designs from the virtual molecular libraries, e.g., based on the similarity to known bioactive molecules, external activity prediction, or reward functions.[^9^, ^13^, ^15^, ^23^] Here, we use the beam search algorithm as a model‐intrinsic alternative to temperature sampling. This method enables the CLM to simultaneously generate and prioritize the molecular designs in an automated fashion, without employing additional selection methods.[^24^, ^25^] Beam search scoring was successfully validated in a prospective application aiming to generate new retinoic acid‐related orphan receptor (ROR)^[26]^ ligands from scratch.

RORs were chosen as molecular targets because these receptor proteins are an attractive but not extensively studied family of potential drug targets. They constitute a family of ligand‐activated transcription factors that mainly act as monomers and are involved in the circadian control of energy homeostasis[^27^, ^28^] and immune system regulation,[^29^, ^30^] among other functions. RORs hold promising pharmacological potential for various indications, specifically for autoimmune diseases.[^29^, ^30^] No ROR ligand has reached drug approval to date, partially owing to compound‐related issues such as poor aqueous solubility, lack of selectivity, and clinical safety concerns.[^29^, ^31^, ^32^]

## Results and Discussion

### Chemical Language Model and Beam Search Sampling for De Novo Design

We explored the beam search algorithm^[33]^ to generate molecules from a CLM as a potential alternative to temperature sampling combined with an external ranking method. Given the probabilities learnt by a CLM, a vast number of SMILES strings could in theory be sampled. As it is computationally not feasible to sample all outputs, a heuristic method such as beam search can be used to find the likely outputs. Here, our underlying hypothesis was that the probability for generating a certain SMILES string correlates with the quality of the corresponding molecule regarding the implicit design objective as represented in the fine‐tuning set (e.g., desired bioactivity, physicochemical properties). During molecule generation by beam search sampling, the algorithm progressively adds tokens to a SMILES string while keeping track of the *k* most likely SMILES string(s). To add a new token, the algorithm computes the conditional probability of each possible token given the tokens in the existing string and defines the *k* most likely tokens to extend the string (Figure 1 c). The set of *k* most likely selections is based on a scoring function (“beam search score”), which is computed as the product of the probabilities of each token (Figure 1 c). This process is repeated until the SMILES string is completed (i.e., the “end‐of‐string” token is added) or a predefined maximal string length is reached. Thus, beam search can be used to generate highly probable molecules, as computed by (i) the underlying model and (ii) the beam search score. The beam search score allows to rank the de novo designs according to the probability of their SMILES tokens.

As a framework to probe beam search sampling, we employed a recently published CLM based on a recurrent neural network with long short‐term memory cells (LSTM), which are suited for sequence modeling.^[34]^ The CLM was trained with the SMILES strings of 365 063 molecules from ChEMBL^[35]^ to iteratively predict the next token of each SMILES string given the preceding tokens (Figure 1 b). The training procedure was carried out over ten epochs, meaning that each molecule used for training was seen by the CLM ten times. This pre‐trained CLM was then fine‐tuned using sets of known ROR ligands (Figure S1, Table S1), to obtain a bias towards the design objective, namely the generation of new molecules with bioactivity on RORs, by transfer learning. Open‐source code for the CLM and the beam search algorithm, and the data used in this study are available at https://github.com/ETHmodlab/molecular design with beam search.

### Application of Beam Search Sampling to Designing Inverse RORγ Agonists

For prospective evaluation, we applied the beam search to the design of natural product‐inspired RORγ ligands. Learning from natural products as a traditional source of inspiration for drug discovery[^36^, ^37^] may hold several advantages over learning from purely synthetic molecules, because of the overall higher structural diversity, greater three‐dimensionality, and often superior selectivity of bioactive natural products.[^38^, ^39^] We aimed to obtain de novo designs possessing three properties: (i) natural product‐inspired chemical structure, (ii) synthesizability, and (iii) bioactivity on RORγ. Aiming to fulfil all three objectives during transfer learning, the previously pre‐trained CLM on bioactive molecules from ChEMBL^[17]^ was fine‐tuned on one synthetic and four natural product RORγ modulators described in literature^[30]^ (Figure S1). From the fine‐tuned model, beam search sampling was started after the fifth epoch of fine‐tuning, to ensure that the CLM had sufficiently captured the molecular features of the small fine‐tuning set.

**Figure 2:**
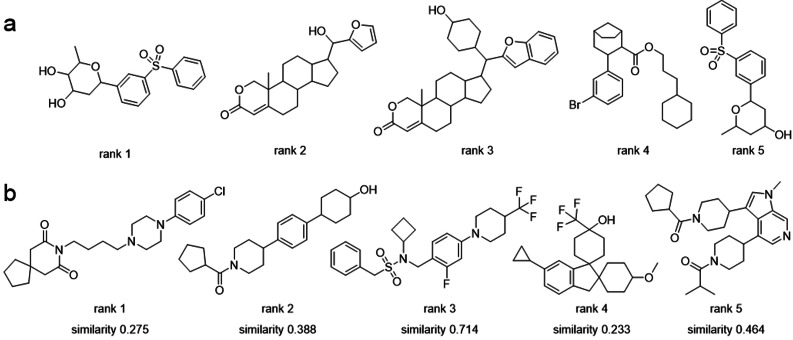
Top‐ranked designs obtained by beam search sampling. a) Single fine‐tuning, b) double fine‐tuning. Ranks are based on the beam search score of the molecular designs. For the top‐ranked molecules from the double fine‐tuning experiment, the similarity values refer to the Tanimoto similarity computed on Morgan fingerprints (length=1024, 2‐bond radius) to the closest known active molecule annotated in ChEMBL with an IC_50_ value for RORγ (structures are shown in Figure S2).

All valid SMILES strings generated between epochs 5 and 16 (last fine‐tuning epoch) were ranked by beam search scoring. The top five designs according to the beam search score (Figure 2 a) were deemed synthetically inaccessible by medicinal chemists. This was further highlighted by the predictions of a machine learning algorithm for retrosynthetic analysis (IBM RXN)^[40]^ which did not find a synthetic route for any of these molecules. Thus, while the CLM captured natural product likeness, the model failed to meet the generic design criterion of synthesizability. These findings point to a benefit of beam search sampling in revealing the most likely CLM molecules to assess the success of fine‐tuning in terms of the design objectives.

**Figure 3:**
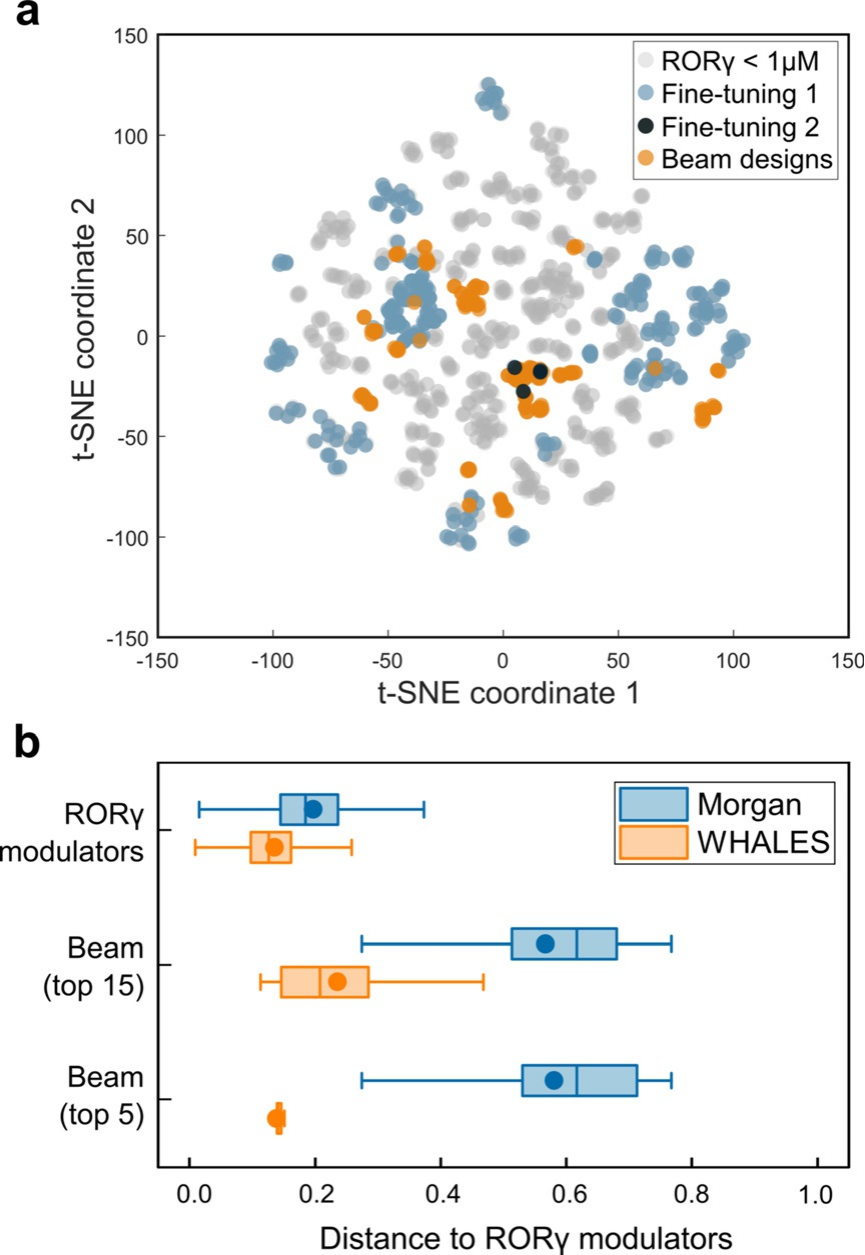
Characteristics of designs from the CLM with double fine‐tuning. a) Stochastic neighbor embedding (t‐SNE)^[45]^ projection of the compound sets based on Morgan fragment fingerprints (length=1024, 2‐bond radius, Tanimoto similarity). The location of the two‐fine tuning sets, the RORγ modulators annotated in ChEMBL (IC_50_<1 μm, 1091 compounds), and the beam search designs are shown. b) Comparison of the sampled molecular designs with known RORγ modulators (IC_50_<1 μm) in terms of Morgan fragment fingerprints (“Morgan”) and three‐dimensional shape and electrostatics descriptors (WHALES). The pairwise distance distribution among known RORγ modulators contained in ChEMBL is shown as a reference. For Morgan fingerprints, the Tanimoto distance is shown; for WHALES the range‐scaled Euclidean distance is shown. “Beam (15)” and “Beam (5)” indicate the top 15 and top 5 designs, respectively. Boxplots indicate 25^th^, 50^th^, and 75^th^ percentiles (lines), mean values (circle), and outlier boundaries (whiskers, 1.5× interquartile range).

Aiming to improve upon these results, we performed a second experiment in which we applied a two‐step fine‐tuning strategy. First, the pre‐trained model was fine‐tuned for 20 epochs on 255 synthetic RORγ ligands from the US patent subset of the Protein Data Bank^[41]^ (255 molecules, Table S1) to capture both bioactivity and synthesizability. Then, the model was fine‐tuned with four natural product RORγ modulators^[30]^ (Figure S1) for 16 epochs, aiming to bias the model towards natural‐product‐likeness. Again, valid SMILES strings generated by beam search sampling between epochs 5 and 16 of the (second) fine‐tuning step were considered. With this second approach, the top 5 sampled molecules (Figure 2 b) were synthetically accessible according to IBM RXN,^[40]^ which could propose a synthetic route for each of them. Importantly, the computer‐generated molecules possess certain natural product characteristics (Figure 3, Table S2), as indicated by a high fraction of sp^3^‐hybridized carbon atoms (Fsp^3^). The top five designs have Fsp^3^ values ranging from 50 % to 75 %. These values are comparable to those computed for the MEGx natural product library (Analyticon Discovery GmbH, rel. 09‐01‐2018), and exceed the average Fsp^3^ value of the ChEMBL molecules used for pre‐training (51±30 % and 33±20 %, respectively). These results suggested that the two‐step fine‐tuning procedure complied with the design objectives and the implemented two‐step approach was chosen for prospective application.

We then compared the beam search designs obtained with the chosen computational strategy to known RORγ modulators and to the fine‐tuning molecules (Figure 3 a,b). Despite favoring only some of the most likely tokens while generating new SMILES strings, and examining only a limited set of possibilities, the beam search sampling still allowed to explore the chemical space beyond the regions that are populated by the fine‐tuning compounds (Figure 3 a). Compared to the inverse RORγ agonists annotated in ChEMBL (IC_50_<1 μm, Figure 2 d), the beam search designs are structurally more diverse in terms of substructure fragments, as represented by Morgan fingerprints.^[42]^ Still, the designs possess a certain degree of similarity to the known active molecules in terms of their three‐dimensional shape and partial charge distribution (as represented by the Weighted Holistic Atom Localization and Entity Shape [WHALES] descriptors[^43^, ^44^]). Apparently, the CLM, in addition to learning the SMILES “syntax”, also learned certain “semantic” ligand features that are relevant for binding to macromolecules, such as their molecular shape and partial charge patterns.

### Prospective Experimental Validation

**Scheme 1:**
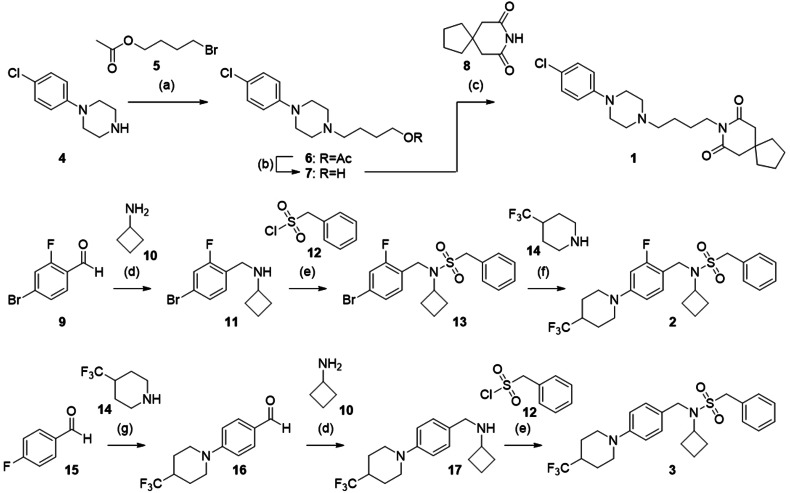
Synthesis of the CLM designs **1**, **2**, and **3**. Reagents and conditions: a) DMF, 4‐DMAP, 60 °C, 16 h, 48 %; b) KOH, H_2_O/THF/MeOH, microwave irradiation, 100 °C, 30 min, 98 %; c) DIAD, PPh_3_, THF, 0 °C→r.t., 16 h, 42 %; d) NaB(OAc)_3_H, HOAc, DCE, r.t., 50 h, 73 %; e) 4‐DMAP, pyridine, CH_2_Cl_2_, reflux, 16 h, 37 %; f) Pd_2_(dba)_3_, xantphos, Cs_2_CO_3_, 1,4‐dioxane, reflux, 16 h, 18 %; g) K_2_CO_3_, DMSO, reflux, 48 h, 82 %.

Three beam search designs were synthesized and characterized in vitro. We selected them based on their beam search score. From the five most likely designs (Figure 2 b), we selected molecules **1** and **2**, which were ranked first and third. Compound **2** showed the highest Tanimoto similarity (Morgan fingerprints) to a known RORγ modulator (Figure 2 b). The scaffolds of both compounds were also prominent among the beam search designs not included in the top 5, suggesting a structural preference. The scaffold of **1** also appeared in the design ranked 6^th^. Molecules ranked 10^th^ and 13^th^ resembled compound **2**. Hence, we additionally chose compound **3** of this abundant chemotype from rank 13 for prospective validation. Compounds **1**–**3** were synthesized according to Scheme 1.

For preparation of **1**, (4‐chlorophenyl)piperazine (**4**) was treated with 4‐bromobutyl acetate (**5**) to obtain the ester‐protected intermediate **6** which after alkaline ester hydrolysis to **7** was suitable for Mitsunobu reaction with 8‐azaspiro[4.5]decane‐7,9‐dione (**8**) to obtain the top‐ranked computational design **1**. Preparation of **2** started from 4‐bromo‐2‐fluorobenzaldehyde (**9**) which was reacted with amine **10** to obtain **11** by reductive amination followed by sulfonamide coupling with **12** to yield **13**. Eventually, the 4‐trifluoromethylpiperidine substituent was introduced to **13** under Buchwald–Hartwig conditions with **14** yielding compound **2**. The structurally related design **3** was prepared via a different route starting from a nucleophilic aromatic substitution of 4‐fluorobenzaldehyde (**15**) with 4‐trifluoromethylpiperidine (**14**) to **16**. The nucleophilic aromatic substitution provided substantially higher yield (see Scheme 1) than the Buchwald–Hartwig reaction but could not be employed in the synthesis of **2** because of the potential formation of regioisomers. Reductive amination of **16** with cyclobutaneamine (**10**) to **17**, followed by sulfonamide formation with phenylmethanesulfonyl chloride (**12**), afforded the computationally designed compound **3**.

In vitro characterization of compounds **1**, **2**, and **3** in Gal4‐ROR hybrid reporter gene assays confirmed inverse RORγ agonism with micromolar to sub‐micromolar IC_50_ values (Table 1). The top‐ranked compound **1** counteracted RORγ activity with an IC_50_ value of 4.6 μm. It was additionally active on RORα and RORβ, but precise IC_50_ values could not be determined due to cytotoxicity. Compounds **2** and **3** blocked RORγ activity with IC_50_ values of 0.37 μm (**2**) and 0.68 μm (**3**), respectively. In addition to being inverse RORγ agonists, all three synthesized designs revealed pronounced preference for the RORγ subtype, with compounds **2** and **3** possessing more than tenfold higher potency on RORγ compared to the related RORα and RORβ isoforms. These results show that the CLM with beam search sampling conserved the bioactivity of the training molecules in the computational designs.

**Table 1:** Activity of de novo designs **1**, **2**, and **3** on RORs in Gal4 hybrid reporter gene assays. Data are reported as mean±S.E.M., *n*≥4.

	IC_50_ [μm]		
Structure and ID	RORα	RORβ	RORγ
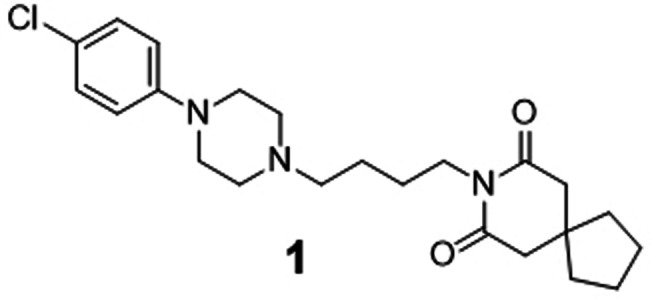	>10	>10	4.6±0.5
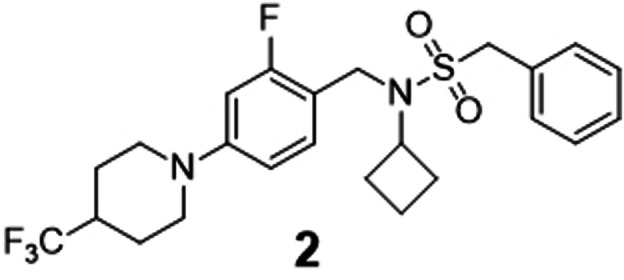	23±3	22±1	0.37±0.05
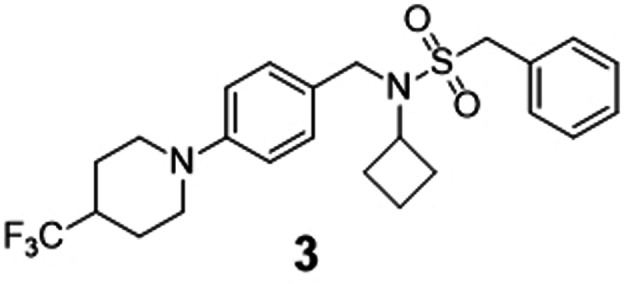	10±1	7.6±0.5	0.68±0.07

## Conclusion

Herein, Beam search sampling from CLMs was applied to generating new molecules with desired bioactivity on the ligand‐activated transcription factor RORγ. The algorithm automatically generated and scored the designs, without the need of additional prioritization rules. Prospective experimental validation yielded three novel, potent inverse agonists of the nuclear receptor with various degrees of similarity to known RORγ modulators (ranging from 0.28 to 0.71, as captured by Tanimoto similarity on Morgan fingerprints). Apparently, the beam search approach coupled with a CLM conserves structural features necessary for the desired bioactivity but still generates structurally diverse compounds in terms of fragments. This observation corroborates beam search sampling as a technique for the de novo design of bioactive molecules by a CLM. The computational and experimental results suggest two attractive properties of the beam search algorithm. Firstly, by searching for the most likely molecules a CLM can generate, the beam search algorithm probes the suitability of a CLM for the given task. Evaluation of the resulting designs allows to check the compliance of the CLM designs with the design objectives and to assess the success of fine‐tuning. This is in contrast to standard temperature sampling, which might lead chemists to consider designs that are not likely according to the model. Secondly, beam search sampling could potentially overcome the need for external compound prioritization. It should be noted, however, that the number of designs that can be sampled by beam search is limited compared to temperature sampling, which can virtually generate an infinite number of chemical structures. The two techniques complement each other, and both offer characteristic advantages. The desired application should guide the choice of either strategy. If corroborated in future prospective studies, beam search sampling may help to further the applicability of CLMs for de novo molecular design in medicinal chemistry.

## Conflict of interest

G.S. declares a potential financial conflict of interest as a founder of inSili.com GmbH, Zurich, and in his role as consultant to the pharmaceutical industry.

## Supporting information

As a service to our authors and readers, this journal provides supporting information supplied by the authors. Such materials are peer reviewed and may be re‐organized for online delivery, but are not copy‐edited or typeset. Technical support issues arising from supporting information (other than missing files) should be addressed to the authors.

Supporting Information
