# Supplementary material for: Beam Search for Automated Design and Scoring of Novel ROR Ligands with Machine Intelligence
Source: Angew Chem Int Ed Engl. 2021 Jul 19;60(35):19477–82. doi: 10.1002/anie.202104405 (PMC8457062; doi:10.1002/anie.202104405)
Supplement: Supplementary file 1 — Supporting Information [file ANIE-60-19477-s001.pdf]

Angewandte Chemie
Eine Zeitschrift der Gesellschaft Deutscher Chemiker

Supporting Information

Beam Search for Automated Design and Scoring of Novel ROR Ligands with Machine Intelligence**

Michael Moret[+], Moritz Helmstädter[+], Francesca Grisoni, Gisbert Schneider, and Daniel Merk**

anie_202104405_sm_miscellaneous_information.pdf

SUPPORTING INFORMATION

Table of Contents

Experimental Procedures 1
Computational Methods 1
Chemistry 3
In vitro Characterization Methods 6
Results & Discussion 7
Analytical data (NMR, HRMS, HPLC) 15
Supplementary References 24

Experimental Procedures

Computational Methods

Open-source access. The code, the data and the procedure to replicate our experiments as well as to run our method on own sets of molecules are available in our GitHub repository (https://github.com/ETHmodlab/molecular_design_with_beam_search).

Data processing. Molecules were encoded as canonical SMILES strings[1] using the RDKit package (v.2018.03, www.rdkit.org) and only SMILES strings with a length of up to 140 characters were retained. SMILES strings were standardized in Python (v3.6.5, www.python.org) by removing stereochemical information, salts and duplicates.

Datasets. We used the processed data we recently published[2] for both ChEMBL (used for pretraining the CLM) and for the representative natural products library, MEGx (released 01 September 2018, Analyticon Discovery GmbH). In total, the processed version of ChEMBL contains 365,063 molecules, and the processed version of MEGx 2,931 molecules. The 255 modulators of RORγ used for double fine-tuning were taken from the US patent subset of the protein data bank (rcsb.org)[3], and the four natural products, along with the synthetic compound were taken from the study of Solt and Burris[4]. The most similar known RORγ modulators with reported IC_{50} were extracted from ChEMBL (organism: *Homo sapiens*).

Chemical language model. We used our recently published framework to implement the CLM[2], which is based on a long short-term memory (LSTM)[5]. The model implementations differed based on the transfer learning strategy used: (i) For single fine-tuning, the model was composed of four layers that have a total of 5,820,515 parameters (layer 1: BatchNormalization, layer 2: LSTM with 1,024 units, layer 3: LSTM with 256 units and layer 4: BatchNormalization). We pretrained the CLM for 10 epochs with a learning rate of 10^{-3} and performed the fine-tuning for 16 epochs with a learning rate of 10^{-4} by keeping layer 2 frozen. (ii) For double fine-tuning, the model was composed of five layers that have a total of 8,444,003 parameters (layer 1: BatchNormalization, layer 2: LSTM with 1,024 units, layer 3: LSTM with 512 units and layer 4: LSTM with 256 units, layer 5: BatchNormalization). We pretrained the CLM for 10 epochs with a learning rate of 10^{-3} and performed two rounds of fine-tuning. The first round was applied for 20 epochs with a learning rate of 10^{-4} by keeping layer 2 frozen. The second round was applied for 20 epochs with a learning rate of 10^{-4} by keeping layer 2 and 3 frozen. The CLMs for both strategies were trained on SMILES strings encoded as one-hot vectors. We used the categorical cross-entropy loss and the Adam optimizer[6]. We applied a 10-fold data augmentation to all molecules.

Beam search ranking. We applied the following procedure for the beam search algorithm:

```
Algorithm 1: Beam search to sample SMILES from a CLM
Result: List of generated SMILES
Beam width = k;
Maximum SMILES length = l;
Initialize list of potential SMILES with starting token: potential_smi = ['G'];
while all SMILES in potential_smi are not complete or number of loop < l do
    for each SMILES in potential_smi do
        for each character in the vocabulary do
            Get probabilities of adding the character to the SMILES by the CLM;
            Create new SMILES by adding the character to the SMILES;
            Compute overall score of the SMILES by multiplying probabilities of each of
              its character;
        end
    end
    Rank list of new SMILES by most likely to less likely according to overall score;
    Prune list of potential SMILES to the k most likely candidates;
    Replace list of potential_smi by the k most likely candidates;
end
```

We used a beam width (k) of 50 and defined the maximum SMILES strings length (l) as 140 tokens. We considered in the ranking molecules sampled from epoch 5 to epoch 16 of fine-tuning; the first epochs were not used to ensure the model was sufficiently biased toward the fine-tuning set.

Stochastic Neighbor Embedding. The t-SNE projection was performed with the "tsne" function of MATLAB R2020a[7] on Morgan Fingerprints ('Distance' = 'jaccard'). The perplexity value was optimized from 5 to 50 with a step equal to 10 to minimize the compression error. A perplexity equal to 5 was chosen, corresponding to the minimum compression error (0.328).

Molecular descriptors. Molecular geometry and the corresponding WHALES descriptors were computed with the code freely available at https://github.com/grisoniFr/scaffold_hopping_whales (v1), with default settings, as previously described[8]. WHALES descriptors were feature standardized with the mean and standard deviation of the background data (known RORγ modulators) and the beam molecules taken together. The Euclidean distance based on WHALES was scaled between 0 and 1 by dividing all distances by the maximum distance computed both on the known RORγ modulators and the beam search molecules. Morgan fingerprints[9] (length=1024, 2-bond radius) were computed using RDKit (v.2018.03) in Python (v3.6.5).

IBM RXN. We used the web interface with default parameters (https://rxn.res.ibm.com/).

PCA plot to highlight natural product likeness. We used the scikit-learn package[10] (v0.23.2) to compute the PCA projection with two parameters (Figure S1). The cumulative explained variance by the two principal components was 85.8%. The descriptors picked (fraction of sp^3-hybridized carbon atoms, polar surface area, number of aliphatic rings containing at least one non-aromatic bond, number of hydrogen bond acceptors and the number of nitrogen and oxygen atoms) were picked for their relevance to compare natural products to synthetic compounds[11,12]. The loadings are reported in Table S3.

Chemistry

General. All chemicals and solvents were of reagent grade and used without further purification unless otherwise specified. All reactions were conducted in oven-dried glassware under argon atmosphere and in absolute solvents. Reactions under microwave irradiation were carried out in a CEM Discover Microwave (CEM GmbH, Kamp-Lintfort, Germany). NMR spectra were recorded on a Bruker AV 500, Bruker AV 400, Bruker AV 300 or a Bruker am250xp spectrometer (Bruker Corporation, Billerica, MA, USA). Chemical shifts (*δ*) are reported in ppm relative to tetramethylsilane (TMS) as reference. Multiplicity is reported: s, singlet; d, doublet; dd, doublet of doublets; t, triplet; td, triplet of doublets; tt, triplet of triplets; m, multiplet. Approximate coupling constants (*J*) are given in hertz (Hz). Mass spectra were obtained on a VG Platform II (Thermo Fischer Scientific, Inc., Waltham, MA, USA) using electrospray ionization (ESI). High-resolution mass spectra were recorded on a MALDI LTQ ORBITRAP XL instrument (Thermo Fisher Scientific). Compounds were purified by preparative HPLC using a Shimadzu preparative LC-20 A Prominence (Shimadzu, Kyoto, Japan) with the following conditions: column, Luna (10 µ C18(2) 100 Å; 250 mm × 21.2 mm; Phenomenex, Torrance, CA, USA); mobile phase, linear gradient from H_2O + 0.1% formic acid/acetonitrile 50:50 to 90:10 within 10 min, 90:10 for 5 min, linear gradient from 90:10 to 50:50 within 1 min and 50:50 for additional 5 min with UV-detection at 245 and 280 nm. Compound purity was analyzed on a Waters 600 Controller HPLC (Waters, Milford, MA, U.S.A.) using a Waters 2487 Dual Absorbance Detector and a Waters 717 plus Autosampler or a VWR Chromaster (VWR, Radnor, PA, U.S.A.) with a 5160 pump system, using a DAD 5430 and 5260 Autosampler both equipped with a MultoHigh100 RP18-5 µ 250×4 mm column (CS-Chromatographie Service GmbH, Langerwehe, Germany) using a gradient (H_2O+0.1 % formic acid/MeOH 80 : 20 isocratic for 5 min to MeOH after additional 45 min and MeOH for additional 10 min) at a flow rate of 1 mL/min or a gradient (H_2O+0.1 % formic acid/MeOH 60 : 40 isocratic for 5 min to MeOH after additional 25 min and MeOH for additional 10 min) at a flow rate of 1 mL/min with UV-detection at 245 nm and 280 nm.

8-(4-(4-(4-Chlorophenyl)piperazin-1-yl)butyl)-8-azaspiro[4.5]decane-7,9-dione (**1**): 4-(4-(4-Chlorophenyl)piperazin-1-yl)butan-1-ol (**7**, 0.14 g, 0.50 mmol, 1.0 eq), 8-azaspiro[4.5]decane-7,9-dione (**8**, 0.092 g, 0.55 mmol, 1.1 eq) and triphenylphosphine (0.14 g, 0.55 mmol, 1.1 eq) were dissolved in THF (10 mL) and the mixture was cooled to 0°C. Diisopropylazodicarboxylate (DIAD, 0.11 g, 0.55 mmol, 1.1 eq) was then slowly added via syringe before the mixture was allowed to warm to room temperature and stirred for 16 h. 1 M sodium hydroxide solution (20 mL) and ethyl acetate (20 mL) were then added, phases were separated, and the aqueous layer was extracted three times with ethyl acetate (3×20 mL). The combined organic layers were dried over Na_2SO_4, and the solvents were evaporated in vacuum. Further purification was performed by column chromatography (*n*-hexane/EtOAc 10:1 + 2% triethylamine) followed by recrystallization from *n*-hexane to obtain **1** as colorless solid (88 mg, 42%). ^{1}H NMR (500 MHz, Methanol-*d$_4$*) δ = 7.23 – 7.19 (m, 2H), 6.97 – 6.92 (m, 2H), 3.82 – 3.78 (m, 2H), 3.34 – 3.32 (m, 4H), 3.22 – 3.16 (m, 4H), 2.65 – 2.62 (m, 4H), 2.49 – 2.42 (m, 2H), 1.77 – 1.72 (m, 4H), 1.60 – 1.50 (m, 8H). ^{13}C NMR (126 MHz, Methanol-*d$_4$*) δ = 173.05, 150.02, 128.46, 124.25, 117.12, 57.86, 52.76, 48.47, 44.07, 39.25, 38.73, 37.03, 25.67, 23.76, 23.50. HRMS (MALDI): *m/z* calculated 418.22558 for $C_{23}H_{33}ClN_3O_2$, found 418.22531 ($[M+H]^+$).

***N*-Cyclobutyl-*N*-(2-fluoro-4-(4-(trifluoromethyl)piperidin-1-yl)benzyl)-1-phenylmethanesulfonamide** (**2**): *N*-(4-Brom-2-fluorbenzyl)-*N*-cyclobutylphenylmethansulfonamide (**13**, 0.100 g, 0.242 mmol, 1.00 eq), 4-(trifluoromethyl)piperidine (**14**, 0.033 mL, 0.242 mmol, 1.00 eq) and caesium carbonate (0.316 g, 970 mmol, 4.00 eq) were suspended in 1,4-dioxane (10 mL). After the addition of 4,5-bis(diphenylphosphino)-9,9-dimethylxanthene (0.024 g, 0.036 mmol, 0.150 eq) and tris(dibenzylideneacetone)dipalladium(0) (0.020 g, 0.022 mmol, 0.090 eq) the resulting reaction mixture was stirred over night at 110°C. After cooling to room temperature, aqueous hydrochloric acid (2 N, 20 mL) was added, phases were separated, and the aqueous layer was extracted with EtOAc (3×20 mL). The combined organic layers were dried over Na_2SO_4, and the solvents were evaporated in vacuum. Further purification was performed by column chromatography (*n*-hexane/EtOAc 10:1) followed by preparative HPLC to obtain **2** as colorless solid (0.021 g, 18%). ^{1}H NMR (500 MHz, Chloroform-*d*) δ = 7.41 – 7.33 (m, 6H), 6.66 (dd, *J* = 8.7, 2.5 Hz, 1H), 6.52 (dd, *J* = 13.5, 2.5 Hz, 1H), 4.16 (s, 2H), 4.14 (s, 2H), 4.12 – 4.06 (m, 1H), 2.70 (td, *J* = 12.5, 2.6 Hz, 2H), 2.17 (m, 1H), 2.08 – 1.99 (m, 2H), 1.98 – 1.86 (m, 4H), 1.77 – 1.65 (m, 2H), 1.63 – 1.33 (m, 4H). ^{13}C NMR (126 MHz, Chloroform-*d*) δ = [161.65, 159.72 (d, *J* = 243 Hz)], [151.95, 151.88 (d, *J* = 10.1 Hz)], 130.79, 130.74, [130.58,128.37, 126.15, 123.94 (q, *J* = 279 Hz)], 129.10, 128.76, 128.69, [115.61, 115.50 (d, *J* = 15.1 Hz)], [112.11, 112.09 (d, *J* = 2.52 Hz)], [102.75, 102.55 (d, *J* = 26.5 Hz)], 58.97, 52.77, 48.22, [41.53, 41.50 (d, *J* = 3.78 Hz)], [40.63, 40.41, 40.19, 39.97 (q, *J* = 27.7 Hz)], 29.39, 24.30, 24.28 (d, *J* = 2.52 Hz), 14.53. HRMS (MALDI): *m/z* calculated 485.18804 for $C_{24}H_{29}F_4N_2O_2S$, found 485.18739 ($[M+H]^+$).

***N*-Cyclobutyl-1-phenyl-N-(4-(4-(trifluoromethyl)piperidin-1-yl)benzyl)methanesulfonamide** (**3**): *N*-(4-(4-(Trifluoromethyl)piperidin-1-yl)benzyl)cyclobutanamine (**17**, 0.104 g, 0.400 mmol, 1.00 eq) was dissolved in methylene chloride (10 mL). After the addition of pyridine (0.032 mL, 0.400 mmol, 1.00 eq), phenylmethanesulfonyl chloride (**12**, 0.077 g, 0.400 mmol, 1.00 eq) and catalytic amounts of 4-(dimethylamino)-pyridine were added. The resulting reaction mixture was stirred over night at 50°C. After cooling to room temperature, aqueous hydrochloric acid (2 N, 20 mL) was added, phases were separated, and the aqueous layer was extracted with EtOAc (3×20 mL). The combined organic layers were dried over Na_2SO_4, and the solvents were evaporated in vacuum. Further purification was performed by column chromatography (*n*-hexane/EtOAc 2:1) to yield **3** as a yellow oil (0.062 g, 37%). ^{1}H NMR (500 MHz, DMSO-*d$_6$*) δ = 7.41 – 7.35 (m, 5H), 7.16 (d, *J* = 8.6 Hz, 2H), 6.91 (d, *J* = 8.7 Hz, 2H), 4.35 (s, 2H), 4.17 (s, 2H), 4.11 – 4.02 (m, 1H), 3.75 (d, *J* = 12.5 Hz, 2H), 2.67 (td, *J* = 12.5, 2.5 Hz, 2H), 2.48 - 2.40 (m, 1H), 2.01 – 1.90 (m, 2H), 1.90 – 1.74 (m, 4H), 1.57 - 149 (m, 2H), 1.46 – 1.30 (m, 2H). ^{13}C NMR (126 MHz, DMSO-*d$_6$*) δ = 149.79, [131.13, 128.92, 126.70, 124.49 (q,

J = 279 Hz)], 130.85, 129.73, 129.19, 128.36, 128.15, 127.95, 115.82, 56.84, 52.06, 47.45, 47.15, [39.17, 38.96, 38.75, 38.54 (q, *J* = 26.5 Hz)], 28.79, 23.80, 23.78, 14.07. HRMS (MALDI): *m/z* calculated 467.19746 for $C_{24}H_{30}F_3N_2O_2S$, found 467.19692 ($[M+H]^+$).

4-(4-(4-Chlorophenyl)piperazin-1-yl)butyl acetate (**6**): *N*-(4-Chlorophenyl)piperazine (**4**, 0.65 g, 3.3 mmol, 1.1 eq), 4-bromobutyl acetate (**5**, 0.59 g, 3.0 mmol, 1.0 eq) and 4-dimethylaminopyridine (1.2 g, 9.0 mmol, 3.0 eq) were dissolved in DMF (20 mL) and the mixture was stirred at 60°C for 16 h. After cooling to room temperature, 1 M sodium hydroxide solution (50 mL) and ethyl acetate (50 mL) were added, phases were separated, and the aqueous layer was extracted three times with ethyl acetate (3×50 mL). The combined organic layers were concentrated in vacuum to approx. 50 mL, and washed twice with 1 M sodium hydroxide solution (2×50 mL). The organic layer was then dried over Na_2SO_4, and the solvents were evaporated in vacuum. Further purification was performed by column chromatography (*n*-hexane/EtOAc 1:1 + 2% triethylamine) to yield **6** as a pale yellow solid (0.45 g, 48%). ^{1}H NMR (500 MHz, Methanol-*d₄*) δ = 7.24 – 7.19 (m, 2H), 6.97 – 6.92 (m, 2H), 4.12 (t, *J* = 6.1, 2H), 3.22 – 3.17 (m, 4H), 2.68 – 2.62 (m, 4H), 2.49 – 2.44 (m, 2H), 2.05 (s, 3H), 1.74 – 1.60 (m, 4H). ^{13}C NMR (126 MHz, Methanol-d_4) δ = 171.57, 150.01, 128.45, 124.25, 117.11, 63.99, 57.75, 52.74, 48.49, 26.33, 22.59, 19.40.

4-(4-(4-Chlorophenyl)piperazin-1-yl)butan-1-ol (**7**): In a microwave tube, 4-(4-(4-chlorophenyl)piperazin-1-yl)butyl acetate (**6**, 0.4 g, 1.3 mmol, 1.0 eq) was dissolved in a mixture of water (2.5 mL), MeOH (2.5 mL) and THF (5.0 mL), potassium hydroxide (0.37 g, 6.5 mmol, 5.0 eq) was added, the tube was sealed and the mixture was stirred under microwave irradiation at 100°C for 30 min. Water (25 mL) and ethyl acetate (25 mL) were added, phases were separated, and the aqueous layer was extracted three times with ethyl acetate (3×30 mL). The combined organic layers were dried over Na_2SO_4, and the solvents were evaporated in vacuum to yield **7** as a colorless solid (0.34 g, 98%) without further purification. ^{1}H NMR (500 MHz, Methanol-d_4) δ = 7.23 – 7.19 (m, 2H), 6.97 – 6.92 (m, 2H), 3.60 (t, *J* = 6.1, 2H), 3.23 – 3.18 (m, 4H), 2.70 – 2.64 (m, 4H), 2.50 – 2.44 (m, 2H), 1.71 – 1.57 (m, 4H). ^{13}C NMR (126 MHz, Methanol-d_4) δ = 149.98, 128.46, 124.29, 117.13, 61.42, 58.12, 52.70, 48.45, 30.55, 22.98.

***N*-(4-Bromo-2-fluorobenzyl)cyclobutylamine** (**11**): 4-Bromo-2-fluorobenzaldehyde (**9**, 0.500 g, 2.46 mmol, 1.00 eq) and cyclobutylamine (**10**, 0.830 mL, 2.71 mmol, 1.10 eq) were dissolved in DCE (10 mL), anhydrous acetic acid (1 mL) was added, and the mixture was stirred at room temperature for 2 h. Sodium triacetoxyborohydride (0.764 g, 3.45 mmol, 1.40 eq) was then added and the mixture was stirred another 48 h at room temperature. Then a saturated solution of sodium bicarbonate (10 mL) was added, phases were separated, and the aqueous layer was extracted with EtOAc (3×20 mL). The combined organic layers were dried over Na_2SO_4, and the solvents were evaporated in vacuum. Further purification was performed by column chromatography (*n*-hexane/EtOAc 1:1 + 2% triethylamine) to yield **11** as a yellow oil (0.463 g, 73%). ^{1}H NMR (400 MHz, DMSO-d_6) δ = 7.47 – 7.32 (m, 3H), 3.58 (s, 2H), 3.11 (p, *J* = 7.3 Hz, 1H), 2.07 - 2.00 (m, 2H), 1.71 – 1.44 (m, 4H). ^{13}C NMR (101 MHz, DMSO-d_6) δ = [161.52, 159.05 (d, *J* = 249 Hz)], [131.95, 131.89 (d, *J* = 6.06 Hz)], [127.71, 127.56 (d, *J* = 15.15 Hz)], [127.27, 127.23 (d, *J* = 4.04 Hz)], [119.58, 119.49 (d, *J* = 9.09 Hz)], [118.31, 118.06 (d, *J* = 25.25 Hz)], 53. 29, 42.69, 30.37, 14.47.

***N*-(4-Bromo-2-fluorobenzyl)-*N*-cyclobutylphenylmethansulfonamide** (**13**): *N*-(4-Bromo-2-fluorobenzyl)cyclobutylamine (**11**, 0.104 g; 0.400 mmol, 1.00 eq) was dissolved in methylene chloride (10 mL). After the addition of pyridine (0.032 mL; 0.400 mmol, 1.00 eq), phenylmethanesulfonyl chloride (**12**, 0.077g; 0.400 mmol; 1.00 eq) and catalytic amounts of 4-(dimethylamino)-pyridine were added. The resulting reaction mixture was stirred over night at 50°C. After cooling to room temperature, aqueous hydrochloric acid (2 N, 20 mL) was added, phases were separated, and the aqueous layer was extracted with EtOAc (3×20 mL). The combined organic layers were dried over Na_2SO_4, and the solvents were evaporated in vacuum. Further purification was performed by column chromatography (*n*-hexane/EtOAc 2:1) to yield **13** as a yellow oil (0.062 g, 37%). ^{1}H NMR (500 MHz, DMSO-d_6) δ = 7.49 (dd, *J* = 9.9, 1.9 Hz, 1H), 7.43 – 7.36 (m, 6H), 7.27 (t, *J* = 8.3 Hz, 1H), 4.46 (s, 2H), 4.29 (s, 2H), 4.21 (tt, *J* = 9.7, 7.6 Hz, 1H), 1.96 – 1.83 (m, 4H), 1.50 – 1.34 (m, 2H). ^{13}C NMR (126 MHz, DMSO-d_6) δ = [160.16, 158.18 (d, *J* = 249 Hz)], 130.98, [130.63, 130.59 (d, *J* = 5.04 Hz], 129.49, 128.54, 128.38, [127.69, 127.66 (d, *J* = 3.78 Hz)], [126.30, 126.19 (d, *J* = 13.9 Hz)], [120.22, 120.14 (d, *J* = 10.1 Hz)], [118.50, 118.30 (d, *J* = 25.2 Hz)], 56.57, 52.06, 40.80, 28.74, 14.04.

4-(4-(Trifluoromethyl)piperidin-1-yl)benzaldehyde (**16**): 4-Trifluoromethylpiperidine (**14**, 1.64 mL, 12.09 mmol, 2.00 eq) and potassium carbonate (4.01 g, 29.01 mmol, 6.00 eq) were suspended in DMSO (15 mL). After the addition of 4-fluorobenzaldehyde (**15**, 0.520 mL, 4.83 mmol; 1.00 eq) the mixture was stirred at 180°C for 48 h. After cooling to room temperature, water (10 mL) was added and the mixture was extracted with EtOAc (5×25 mL). The combined organic layers were dried over Na_2SO_4, and the solvents were evaporated in vacuum. Further purification was performed by column chromatography (*n*-hexane/EtOAc 3:1) to yield **16** as a yellow solid (1.02 g, 82%). ^{1}H NMR (400 MHz, Methanol-d_4) δ = 9.68 (s, 1H), 7.76-7.73 (m, 2H), 7.05-7.02 (m, 2H), 4.12 (d, *J* = 12.8 Hz, 2H), 2.96 (t, *J* = 12.8 Hz, 2H), 2.54 – 2.38 (m, 1H), 1.96 (d, *J* = 13.0 Hz, 2H), 1.62 (q, *J* = 12.6 Hz, 2H). ^{13}C NMR (101 MHz, Methanol-d_4) δ = 182.97, 147.00, 123.69, 118.58, 105.46, 91.93, 37.94, [32.29, 32.02, 31.74, 31.48 (q, *J* = 27.27 Hz)], [15.71, 15.69, 15.66, 15.63 (q, *J* = 3.03 Hz)].

***N*-(4-(4-(Trifluoromethyl)piperidin-1-yl)benzyl)cyclobutaneamine** (**17**): 4-(4-(Trifluoromethyl)piperidin-1-yl)benzaldehyde (**16**, 0.500 g, 1.94 mmol, 1.00 eq) and cyclobutylamine (**10**, 0.182 mL, 2.14 mmol, 1.10 eq) were dissolved in DCE (5 mL), anhydrous acetic acid (0.3 mL) was added, and the mixture was stirred at room temperature for 2 h. Sodium triacetoxyborohydride (0.577 g, 2.72 mmol, 1.40 eq) was then added and the mixture was stirred another 48 h at room temperature. Then a saturated solution of sodium bicarbonate (10 mL) was added, phases were separated, and the aqueous layer was extracted with EtOAc (3×15 mL). The

combined organic layers were dried over Na_2SO_4, and the solvents were evaporated in vacuum. Further purification was performed by column chromatography (*n*-hexane/EtOAc 1:1 + 2% triethylamine) to yield **17** as a yellow oil (0.463 g, 73%). ^{1}H NMR (400 MHz, DMSO-d_6) δ = 7.17 – 7.10 (m, 2H), 6.90 – 6.84 (m, 2H), 3.74 – 3.70 (m, 2H), 3.48 (s, 2H), 3.11 (p, *J* = 7.3 Hz, 1H), 2.66 (td, *J* = 12.5, 2.5 Hz, 2H), 2.13 – 1.93 (m, 3H), 1.88 – 1,84 (m, 2H), 1.72 – 1.44 (m, 6H). ^{13}C NMR (126 MHz, DMSO-d_6) δ = 149.53, 131.68, [131.15, 128.93, 126.72, 124.51 (q, *J* = 279 Hz)], 128.65, 115.91, 53.16, 49.53, 47.82, [39.21, 39.00, 38.79, 38.58 (q, *J* = 26.5 Hz)], 30.43, [23.82, 23.80, 23.78, 23.76 (q, *J* = 2,5 Hz)], 14.51.

In vitro Characterization Methods

Hybrid reporter gene assays. The hybrid receptor plasmids pFA-CMV-hRORα-LBD (NR1F1, isoform 1, uniprot ID P35398-2, aa 266-523), pFA-CMV-hRORβ-LBD (NR1F2, isoform 2, uniprot ID Q92753-2, aa 216-470), and pFA-CMV-hRORγ-LBD (NR1F3, isoform 1, uniprot ID P51449, aa 263-518) encode the Gal4-DBD fused to the hinge region and ligand binding domain of the canonical isoform of the respective human nuclear receptor and were cloned as described previously[13,14] using cDNA from SourceBioScience (Nottingham, UK). The Gal4-responsive firefly luciferase reporter pFR-Luc (Stratagene, La Jolla, CA, USA) and pRL-SV40 (Promega, Madison, WI, USA; internal control) were used together with either pFA-CMV-NR-LBD clone for the hybrid reporter gene assays. HEK293T cells (German Collection of Microorganisms and Cell Culture GmbH, DSMZ) were cultured in Dulbecco's modified Eagle's medium (DMEM), high glucose supplemented with 10 % fetal calf serum (FCS), sodium pyruvate (1 mM), penicillin (100 U/mL), and streptomycin (100 µg/mL) at 37 °C and 5 % CO_2 and seeded in 96-well plates (3×10^4 cells/well) twenty-four hours prior to transfection. Before transfection, medium was changed to Opti-MEM without supplements and transient transfection with above mentioned plasmids (pFR-Luc, pRL-SV40 and one pFA-CMV-NR-LBD clone) was carried out with Lipofectamine LTX reagent (Invitrogen) according to the manufacturer's protocol. Five hours after transfection, medium was changed to Opti-MEM supplemented with penicillin (100 U/mL) and streptomycin (100 µg/mL) additionally containing 0.1 % DMSO and the respective test compound or 0.1 % DMSO alone as untreated control. Each concentration was tested in duplicates and each experiment was repeated independently at least two times. Following overnight (14−16 h) incubation, cells were assayed for luciferase activity using the Dual-Glo Luciferase Assay System (Promega) according to the manufacturer's protocol. Luminescence was measured with a Tecan Spark luminometer (Tecan Deutschland GmbH, Germany). Normalization of transfection efficiency and cell growth was done by division of firefly luciferase data by Renilla luciferase data and multiplying the value by 1000 resulting in relative light units (RLU). Fold reporter repression was obtained by dividing the mean RLU of a test compound at a respective concentration by the mean RLU of the 0.1% DMSO control. IC_{50} values were obtained by plotting fold reporter activation vs test compound concentrations and fitting the resulting sigmoidal curve with a four-parameter logistic regression in SigmaPlot 12.5 (Systat Software GmbH, Erkrath, Germany). The Gal4-ROR hybrid assays were validated with the reference inverse agonists SR1001 and SR1078, which yielded IC_{50} values in agreement with the literature.

Results and Discussion

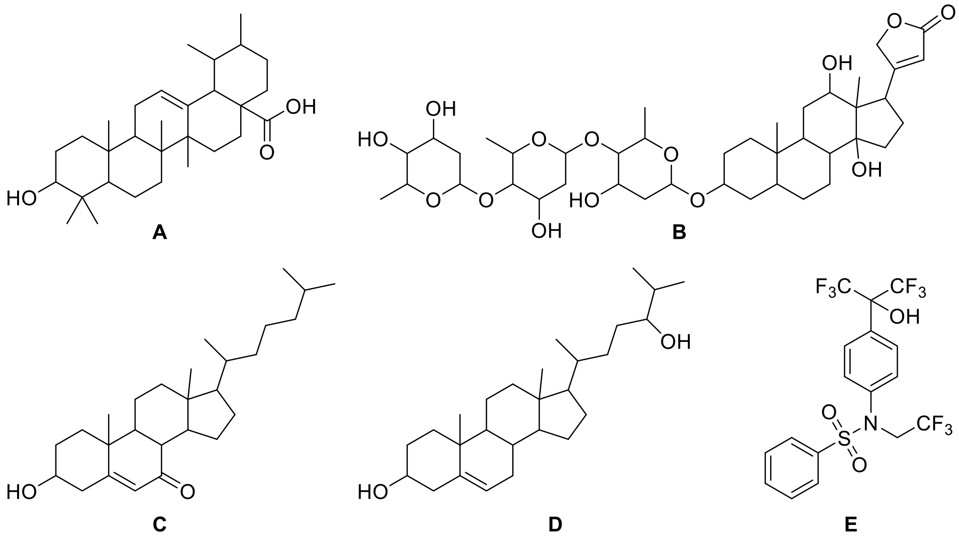

Figure S1 | ROR modulators (natural products **A**-**D** and synthetic compound **E**) used for fine-tuning. **A**-**E** were used for the first fine-tuning approach. **A**-**D** were used in the second stage of the second fine-tuning approach.

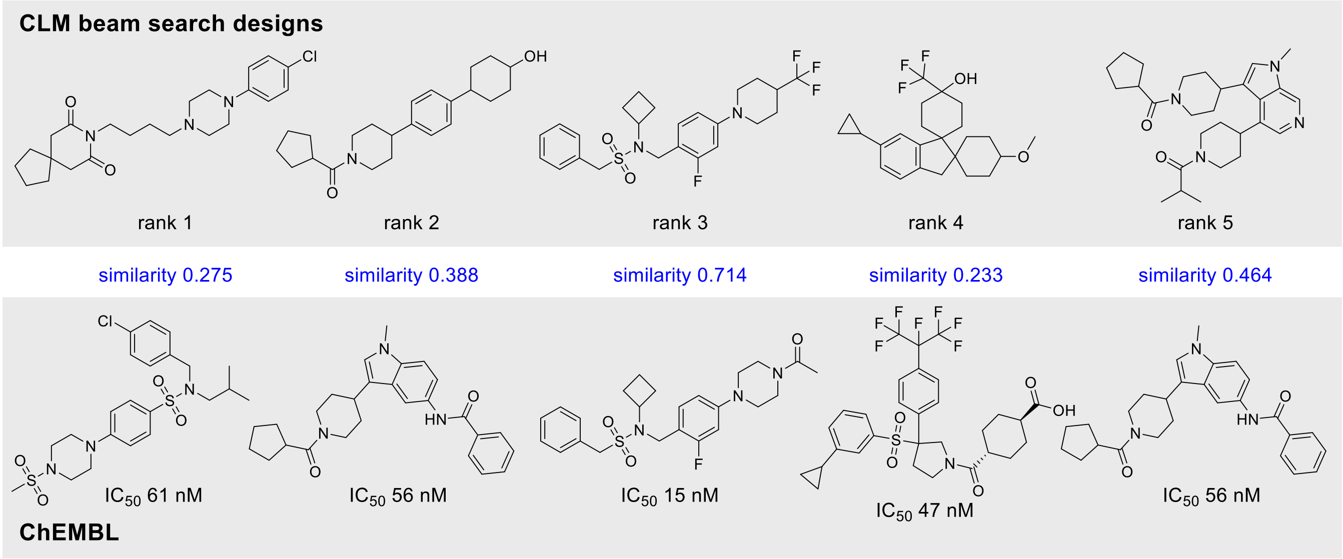

Figure S2 | Five top ranked beam search sampling designs from the second fine-tuning strategy and the corresponding most similar RORγ ligands annotated in ChEMBL with their reported IC_{50} (lowest value if more than one was reported). The similarity values refer to the Jaccard-Tanimoto similarity computed on Morgan fingerprints (length=1024, 2-bond radius).

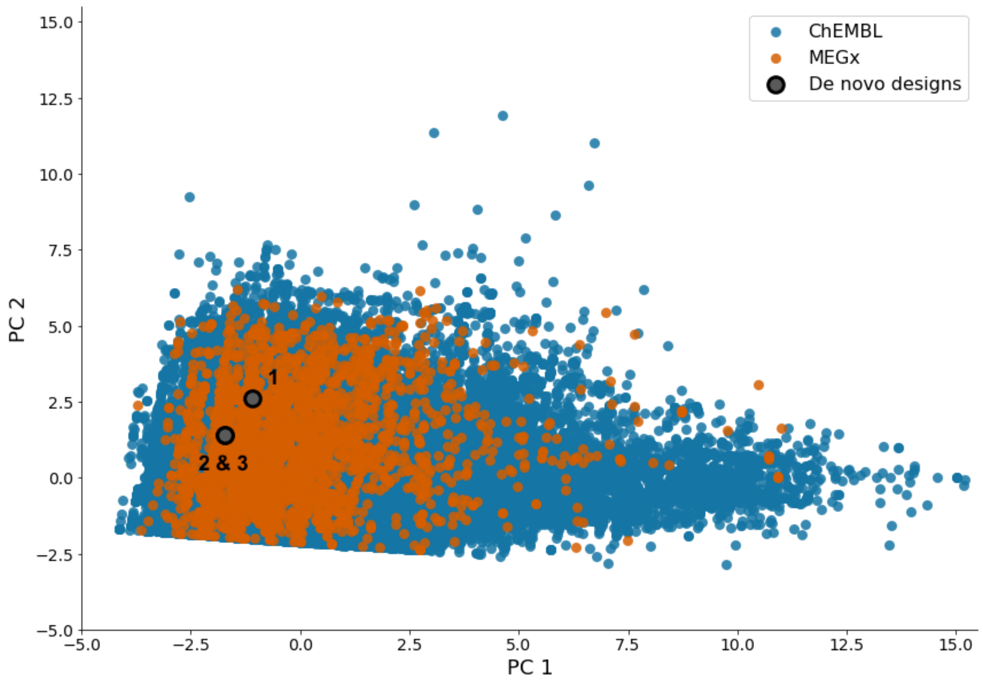

Figure S3 | Principal Component Analysis based on descriptors known to differ between synthetic molecules and natural products. In blue, molecules from ChEMBL. In orange, molecules for the natural products library MEGx. In grey, with thin black lines, de novo designs **1**, **2** and **3.**

Table S1 | Synthetic ROR modulators used for fine-tuning (in the first stage of the second fine-tuning approach) represented as SMILES strings.

COCCOc1ccc(C(=O)Nc2cnc3c(c(C4CCN(C(=O)C(C)C(C)(C)C)CC4)cn3C)c2C)cc1C#N
NC(=O)C1CCN(c2ccc(CN(CC(F)(F)F)S(=O)(=O)Cc3ccccc3)c(F)c2)CC1
CC(=O)N1CCN(c2ccc(CN(C3CCC3)S(=O)(=O)c3ccccc3C#N)c(F)c2)CC1
CC(=O)N1CCN(c2ccc(CN(C3CCC3)S(=O)(=O)CCc3cccc(F)c3)c(F)c2)CC1
CC(C)CN(Cc1ccc(N2CCCS(=O)(=O)CC2)cc1)S(=O)(=O)Cc1ccccc1
CC(C)CN(c1cccc(Cl)c1)S(=O)(=O)c1ccc(-c2ccc(C(N)=O)cc2)cc1
CC(=O)N1CCN(c2ccc(CN(C3CCC3)S(=O)(=O)c3ccc(C(C)C)cc3)c(F)c2)CC1
O=S1(=O)CCN(c2ccc(CN(CC(F)(F)F)S(=O)(=O)Cc3ccccc3)c(F)c2)CC1
CC(=O)N1CCN(c2ccc(CN(C3CCC3)S(=O)(=O)c3ccc(C(F)(F)F)cc3)c(F)c2)CC1
Cc1c(NC(=O)c2cccc(C#N)c2)cnc2c1c(C1CCN(C(=O)C3CCCC3C(C)C)CC1)cn2C
COc1ccc(C#N)cc1C(=O)Nc1cnc2c(c(C3CCN(C(=O)C(C)C(C)(C)C)CC3)cn2C)c1C
COc1cc(C#N)cc(C(=O)Nc2cnc3c(c(C4CCN(C(=O)C5CCCC5)CC4)cn3C)c2C)c1
COCC1CCN(c2ccc(CN(CC(C)C)S(=O)(=O)Cc3ccccc3)cc2)CC1
CC(C)CN(Cc1ccc(N(C)C2CCN(S(C)(=O)=O)CC2)cc1)S(=O)(=O)Cc1ccccc1
CC(C)CN(c1cccc(Cl)c1)S(=O)(=O)c1cccc(-c2ccc(S(C)(=O)=O)cc2)c1
Cc1c(NC(=O)c2cccc(C#N)c2)cnc2c1c(C1CCN(C(=O)C(C)C(C)C)CC1)cn2C
CC(C)C(=O)N1CCC(c2cn(C)c3ncc(NC(=O)c4ccc(Cl)c(C#N)c4)c(C(F)(F)F)c23)CC1
COc1cccc(S(=O)(=O)N(Cc2ccc(N3CCN(C(C)=O)CC3)cc2F)C2CCC2)c1
CC(C)CN(Cc1ccc(NC2CCN(C(=O)N(C)C)CC2)cc1)S(=O)(=O)Cc1ccccc1
Cc1c(NC(=O)c2cccc(C#N)c2)cnc2c1c(C1CCN(C(=O)CC3CCC3)C(C)(C)C1)cn2C
CC(=O)N1CCN(c2ccc(CN(C3CCC3)S(=O)(=O)c3ccc(Cl)cc3)c(F)c2)CC1
COc1cc(Cl)cc(C(=O)Nc2cnc3c(c(C4CCN(C(=O)C(C)C(C)(C)C)CC4)cn3C)c2C)c1
CC(C)CN(Cc1ccc(N2CCC(C#N)C2)cc1)S(=O)(=O)Cc1ccccc1
CC(C)CN(Cc1ccc(N2CCC(C(N)=O)CC2)cc1)S(=O)(=O)Cc1ccccc1
Cc1c(NC(=O)c2cccc(C#N)c2)cnc2c1c(C1CCN(C(=O)C3CCCC3C)CC1)cn2C
Cc1c(NC(=O)c2ccc(F)c(C#N)c2)cnc2c1c(C1CCN(C(=O)C3CCCC3)C(C)(C)C1)cn2C
Cc1c(NC(=O)c2cccc(C#N)c2)cnc2c1c(C1CCN(C(=O)C(C)C)C(C)C1)cn2C
CC(C)CN(Cc1ccc(NC2CCN(S(C)(=O)=O)CC2F)cc1)S(=O)(=O)Cc1ccccc1
CC(C)CN(C(C)c1ccc(N2CCC(O)CC2)cc1)S(=O)(=O)Cc1ccccc1
CC(C)CN(C(C)c1ccc(N2CCN(S(=O)(=O)N(C)C)CC2)cc1)S(=O)(=O)Cc1ccccc1
COc1ccc(C(=O)Nc2cnc3c(c(C4CCN(C(=O)C(C)C(F)(F)F)C(C)C4)cn3C)c2C)cc1C#N
CC(C)CN(Cc1ccc(N2CCC(O)C2)cc1)S(=O)(=O)Cc1ccccc1
CC1CCCC1C(=O)N1CCC(c2cn(C)c3ncc(NC(=O)c4cccc(C#N)c4)c(C(F)(F)F)c23)CC1
COc1cc(Cl)cc(C(=O)Nc2cnc3c(c(C4CCN(C(=O)C(C)C)C(C)(C)C4)cn3C)c2C)c1
COc1ccc(C(=O)Nc2cnc3c(c(C4C5CCC4CN(C(=O)C4CCCC4)C5)cn3C)c2C)cc1C#N
CC(C)CN(Cc1ccc(OC2CC3CCC(C2)N3S(C)(=O)=O)cc1)S(=O)(=O)Cc1ccccc1
CC(C)CN(Cc1ccc(OC2CCN(S(C)(=O)=O)C2)cc1)S(=O)(=O)Cc1ccccc1
CC(C)CN(Cc1ccc(N2CCC(O)CC2)cc1)S(=O)(=O)Cc1ccccc1
COCC(=O)N1CCN(c2ccc(CN(CC(C)C)S(=O)(=O)Cc3ccccc3)cc2)CC1
CC(=O)N1CCN(c2ccc(CN(C3CCC3)S(=O)(=O)c3cccc(C(F)(F)F)c3)c(F)c2)CC1
CC(C)CN(Cc1ccc(N2CCC(N3CCS(=O)(=O)CC3)CC2)cc1)S(=O)(=O)Cc1ccccc1
CC(=O)N1CCN(c2ccc(CN(C3CCC3)S(=O)(=O)c3c(Cl)cccc3Cl)c(F)c2)CC1
CC(C)CN(Cc1ccc(N2CCN(C(=O)C3(C)COC3)CC2)cc1)S(=O)(=O)Cc1ccccc1
CC(C)C(=O)N1CCC(c2cn(C)c3ncc(NC(=O)c4cccc(C#N)c4)c(C(F)(F)F)c23)CC1
CC(C)CN(Cc1ccc(N2CCN(CCO)CC2)cc1)S(=O)(=O)Cc1ccccc1
CC(C)CN(Cc1ccc(N2CCC(S(=O)(=O)N(C)C)CC2)cc1)S(=O)(=O)Cc1ccccc1
CCN(Cc1ccc(N2CCN(C(C)=O)CC2)cc1)S(=O)(=O)Cc1ccccc1
Cn1cc(C2CCN(C(=O)C3CCCC3)CC2)c2c(C(F)(F)F)c(NC(=O)c3ccc(F)c(C#N)c3)cnc21
COc1cc(Cl)cc(C(=O)Nc2cnc3c(c(C4CCN(C(=O)C5CCCC5)C(C)(C)C4)cn3C)c2C)c1
Cc1c(NC(=O)c2cccc(C#N)c2)cnc2c1c(C1CCN(C(=O)C(C)C(F)(F)F)CC1)cn2C
COCCOc1cc(C#N)cc(C(=O)Nc2cnc3c(c(C4CCN(C(=O)C5CCCC5)CC4)cn3C)c2C)c1
CC(=O)N1CCN(c2ccc(CN(C)S(=O)(=O)Cc3ccccc3)cc2)CC1
CC(=O)N1CCN(c2ccc(C(C)N(CC(C)C)S(=O)(=O)Cc3ccccc3)cc2)CC1

```
CC(C)CN(Cc1ccc(N2CCN(C(=O)C(C)O)CC2)cc1)S(=O)(=O)Cc1ccccc1
CC(C)C(C)(C)CC(=O)N1CCC(c2cn(C)c3ncc(NC(=O)c4cccc(C#N)c4)c(C(F)(F)F)c23)CC1
COc1ccc(C(=O)Nc2cnc3c(c(C4CCN(C(=O)C(C)C(C)(C)C)CC4)cn3C)c2C(F)(F)F)cc1C#N
COc1cc(C#N)cc(C(=O)Nc2cnc3c(c(C4CCN(C(=O)C5CCCC5)CC4)cn3C)c2C(F)(F)F)c1
CS(=O)(=O)N1CCN(c2ccc(CN(CC(F)(F)F)S(=O)(=O)Cc3ccccc3)c(F)c2)CC1
CC(=O)N1CCN(c2ccc(CN(CC(F)(F)F)S(=O)(=O)C(C)c3ccccc3)c(F)c2)CC1
CC(C)CN(c1ccccc1Cl)S(=O)(=O)c1ccc(-c2ccc(NS(C)(=O)=O)cc2)cc1
CC(=O)N1CCC2(CCN(c3ccc(CN(CC(C)C)S(=O)(=O)Cc4ccccc4)cc3)C2)C1
Cc1c(NC(=O)c2cc(O)cc(C#N)c2)cnc2c1c(C1CCN(C(=O)C3CCCC3)C(C)(C)C1)cn2C
CC(C)CN(Cc1ccc(NC2CCOCC2)cc1)S(=O)(=O)Cc1ccccc1
CC(=O)N1CCN(c2cnc(CN(CC(C)C)S(=O)(=O)Cc3ccccc3)nc2)CC1
CC(=O)N1CCN(c2ccc(CN(C3CCC3)S(=O)(=O)c3ccc(F)cc3F)c(F)c2)CC1
Cn1cc(C2CCN(C(=O)C3CCCC3)CC2)c2c(C(F)(F)F)c(NC(=O)c3cc(O)cc(C#N)c3)cnc21
CC(=O)N1CCN(c2ccc(CN(C3CCC3)S(=O)(=O)c3ccccc3C)c(F)c2)CC1
CC(=O)N1CCN(c2ccc(CN(C3CCC3)S(=O)(=O)CCc3ccc(F)cc3)c(F)c2)CC1
Cc1c(NC(=O)c2cc(Cl)ccc2F)cnc2c1c(C1CCN(C(=O)C(C)C(C)(C)C)CC1)cn2C
Cc1c(NC(=O)c2cccc(C#N)c2)cnc2c1c(C1CCN(C(=O)CC3CCCCC3)C(C)(C)C1)cn2C
CC(=O)N1CCN(c2ccc(CN(C3CCC3)S(=O)(=O)Cc3ccccc3)cc2)CC1
CC(=O)Nc1ccc2c(c1)CCC(=O)N2Cc1ccc(C(O)(C(F)(F)F)C(F)(F)F)cc1
Cc1c(NC(=O)c2cccc(C#N)c2)cnc2c1c(C1CCN(C(=O)C(C)C(F)(F)F)C(C)C1)cn2C
Cc1c(NC(=O)c2cccc(C#N)c2)cnc2c1c(C1CCN(C(=O)C3CC4CCC3C4)CC1)cn2C
COc1ccc(C(=O)Nc2cnc3c(c(C4CCN(C(=O)C(C)C)CC4C)cn3C)c2C)cc1C#N
COc1ccccc1S(=O)(=O)N(Cc1ccc(N2CCN(C(C)=O)CC2)cc1F)C1CCC1
CC(C)CN(c1ccccc1Cl)S(=O)(=O)c1ccc(-c2ccc(S(N)(=O)=O)cc2)cc1
CC(C)CN(Cc1ccc(N2CCC(C(N)=O)C2)cc1)S(=O)(=O)Cc1ccccc1
CC(C)CN(c1ccccc1C(F)(F)F)S(=O)(=O)c1ccc(-c2ccc(S(C)(=O)=O)cc2)cc1
CC(=O)N1CCN(c2ccc(CN3CCCN(c4ccccc4)S3(=O)=O)c(F)c2)CC1
CC(C)CN(Cc1ccc(N2CCN(C(=O)C3(O)CC3)CC2)cc1)S(=O)(=O)Cc1ccccc1
CC(=O)N1CCN(c2ncc(CN(CC(C)C)S(=O)(=O)Cc3ccccc3)cn2)CC1
CC(=O)N1CCN(c2ccc(CN(C3CCC3)S(=O)(=O)Cc3ccc(F)cc3F)c(F)c2)CC1
COc1cc(Cl)cc(C(=O)Nc2cnc3c(c(C4CCN(C(=O)C(C)C)CC4)cn3C)c2C(F)(F)F)c1
COc1ccc(S(=O)(=O)N(Cc2ccc(N3CCN(C(C)=O)CC3)cc2F)C2CCC2)cc1
CC(C)CN(Cc1ccc(N2CC(O)C2)cc1)S(=O)(=O)Cc1ccccc1
CC(C)CN(Cc1ccc(N2CCC(C(C)(C)O)CC2)cc1)S(=O)(=O)Cc1ccccc1
CC(C)CN(Cc1ccc(N2CCC3(CCNC3=O)C2)cc1)S(=O)(=O)Cc1ccccc1
CC(=O)N1CCN(c2ccc(CN(C3CCC3)S(=O)(=O)c3ccc(Cl)c(Cl)c3)c(F)c2)CC1
CC(=O)N1CCN(c2ccc(CN(C3CCC3)S(=O)(=O)Cc3ccc(Cl)cc3Cl)c(F)c2)CC1
Cn1cc(C2CCN(C(=O)C3CCCC3)C(C)(C)C2)c2c(C(F)(F)F)c(NC(=O)c3cccc(C#N)c3)cnc21
COc1ccc(C(=O)Nc2cnc3c(c(C4CCN(C(=O)C(C)C)CC4)cn3C)c2C(F)(F)F)cc1C#N
CC(C(=O)N1CCC(c2cn(C)c3ncc(NC(=O)c4cccc(C#N)c4)c(C(F)(F)F)c23)CC1)C(C)(C)C
CC1CN(C(=O)C2CCCC2)CCC1c1cn(C)c2ncc(NC(=O)c3cccc(C#N)c3)c(C(F)(F)F)c12
CC(=O)N1CCC(c2ccc(CN(CC(F)(F)F)S(=O)(=O)Cc3ccccc3)c(F)c2)CC1
CC(=O)N1CCN(c2ccc(CN(C3CCC3)S(=O)(=O)Cc3ccccc3)cc2F)CC1
CC(C)CN(Cc1ccc(N2CC3COCC3C2)cc1)S(=O)(=O)Cc1ccccc1
CC(C)CN(Cc1ccc(N2CC(C3CCOCC3)C2)cc1)S(=O)(=O)Cc1ccccc1
CC(C)CC(=O)N1CCC(c2cn(C)c3ncc(NC(=O)c4cccc(C#N)c4)c(C(F)(F)F)c23)CC1
COc1ccc(C(=O)Nc2cnc3c(c(C4CCN(C(=O)C(C)C)CC4)cn3C)c2C)cc1C#N
CC(=O)N1CCN(c2ccc(CN3C(C)CCN(c4ccccc4)S3(=O)=O)c(F)c2)CC1
CC(C)CN(Cc1ccc(N2CCOCC2)cc1)S(=O)(=O)Cc1ccccc1
CC(C)CN(Cc1ccc(N2CCNC(=O)C2)cc1)S(=O)(=O)Cc1ccccc1
CC(C)CN(Cc1ccc(NC2CCN(S(C)(=O)=O)CC2)cc1)S(=O)(=O)Cc1ccccc1
CC(=O)N1CCN(c2ccc(CN(CC(C)C)S(=O)(=O)Cc3ccccc3)nc2)CC1
CC(=O)N1CCN(c2ccc(CN(C3CCC3)S(=O)(=O)c3cc(F)ccc3F)c(F)c2)CC1
CC(=O)N1CCN(c2ccc(CN(C3CCC3)S(=O)(=O)c3cc(Cl)ccc3Cl)c(F)c2)CC1
CC(=O)N1CCN(c2ccc(CN(C3CCC3)S(=O)(=O)c3ccc(OC(F)(F)F)cc3)c(F)c2)CC1
COc1ccc(C(=O)Nc2cnc3c(c(C4C5CCC4CN(C(=O)C(C)C)C5)cn3C)c2C(F)(F)F)cc1C#N
COCCN(Cc1ccc(N2CCN(C(C)=O)CC2)cc1)S(=O)(=O)Cc1ccccc1
```

Cn1cc(C2CCN(C(=O)C3CCCCC3)CC2)c2c(C(F)(F)F)c(NC(=O)c3cccc(C#N)c3)cnc21
CC(=O)N1CC2(C1)CN(c1ccc(CN(CC(C)C)S(=O)(=O)Cc3ccccc3)cc1)C2
CC(=O)N1CCN(c2ccc(CN3CCCCN(c4ccccc4)S3(=O)=O)c(F)c2)CC1
CC(=O)N1CCN(c2ccc(CN(C3CCC3)S(=O)(=O)c3cccc(C(C)(C)C)c3)c(F)c2)CC1
CCOC(=O)N1CCN(c2ccc(CN(CC(C)C)S(=O)(=O)Cc3ccccc3)cc2)CC1
CC(=O)N1CCN(c2ccc(CN(CC(F)(F)F)S(=O)(=O)Cc3ccccc3)c(F)c2)CC1
Cc1c(NC(=O)c2cccc(C#N)c2)cnc2c1c(C1CCN(C(=O)C(C)C)C3(CC3)C1)cn2C
CC(=O)N1CCN(c2ccc(CN(CC(C)C)S(=O)(=O)Cc3ccccc3)cc2)CC1
CC(=O)N1CCN(c2ccc(CN(C3CCC3)S(=O)(=O)Cc3ccccc3)c(F)c2)CC1
CC(=O)N1CCN(c2ccc(CN(C3CCC3)S(=O)(=O)c3cc(F)cc(F)c3)c(F)c2)CC1
Cc1ccc(C(=O)Nc2cnc3c(c(C4CCN(C(=O)C(C)C)CC4)cn3C)c2C(F)(F)F)cc1C#N
Cc1c(NC(=O)c2cccc(C#N)c2)cnc2c1c(C1CCN(C(=O)CC(O)C(F)(F)F)C(C)(C)C1)cn2C
CC(C)CN(Cc1ccc(N2CCCC2)cc1)S(=O)(=O)Cc1ccccc1
CC(=O)N1CCN(c2ccc(CN(C3CCC3)S(=O)(=O)CCc3ccccc3)c(F)c2)CC1
Cc1c(NC(=O)c2cccc(C#N)c2)cnc2c1c(C1CCN(C(=O)C(C)C)CC1C)cn2C
Cc1c(NC(=O)c2cccc(C#N)c2)cnc2c1c(C1CCN(C(=O)C(C)C(F)(F)F)CC1C)cn2C
CN(C)S(=O)(=O)N1CCN(c2ccc(CN(CC(F)(F)F)S(=O)(=O)Cc3ccccc3)c(F)c2)CC1
CC(=O)N1CCN(c2ccc(CN(C3CCC3)S(=O)(=O)c3cc(Cl)cc(Cl)c3)c(F)c2)CC1
CC(=O)N1CCN(c2ccc(CN(C3CCC3)S(=O)(=O)c3cccc(OC(F)F)c3)c(F)c2)CC1
CC(=O)N1CCN(c2ccc(CN(C3CCC3)S(=O)(=O)c3ccccc3)c(F)c2)CC1
CC(=O)N1CCN(c2ccc(CN(C3CCC3)S(=O)(=O)c3ccccc3Cl)c(F)c2)CC1
CC(=O)N1CCN(c2ccc(CN(CC(C)C)S(=O)(=O)Cc3ccccc3)c(C#N)c2)CC1
CC(C)CN(Cc1ccc(NC2CCC(CC#N)C2)cc1)S(=O)(=O)Cc1ccccc1
CC(C)CN(Cc1ccc(N2CC(C(=O)N(C)C)C2)cc1)S(=O)(=O)Cc1ccccc1
CC(C)CN(Cc1ccccc1)S(=O)(=O)c1ccc(-c2ccc(NS(C)(=O)=O)cc2)cc1
CC(C)CN(Cc1ccc(N2CCN(S(=O)(=O)N(C)C)CC2)cc1)S(=O)(=O)Cc1ccccc1
CC(C)C(=O)N1CCC(c2cn(C)c3ncc(NC(=O)c4ccc(F)c(C#N)c4)c(C(F)(F)F)c23)CC1
Cc1cccc(F)c1C(=O)N1CCC(c2cn(C)c3ncc(NC(=O)c4cccc(C#N)c4)c(C)c23)CC1
CC(=O)N1CCN(c2ccc(CN(C3CCC3)S(=O)(=O)c3ccccc3F)c(F)c2)CC1
CC(C)CN(Cc1ccc(N2CCN(C(=O)C3CC3)CC2)cc1)S(=O)(=O)Cc1ccccc1
CC(C)CN(c1ccccc1Cl)S(=O)(=O)c1ccc(-c2ccc(C(N)=O)cc2)cc1
COc1cc(C#N)cc(C(=O)Nc2cnc3c(c(C4CCN(C(=O)C5CCCC5)C(C)(C)C4)cn3C)c2C)c1
CC(=O)N1CCN(c2ccc(CN(C3CCC3)S(=O)(=O)c3c(F)cccc3F)c(F)c2)CC1
CC(=O)N1CCC2(CC1)CN(c1ccc(CN(CC(C)C)S(=O)(=O)Cc3ccccc3)cc1)C2
Cn1cc(C2CCN(C(=O)CC3CC3)CC2)c2c(C(F)(F)F)c(NC(=O)c3cccc(C#N)c3)cnc21
CC(=O)N1CCN(c2ccc(CN(C3CCC3)S(=O)(=O)Cc3cc(F)ccc3F)c(F)c2)CC1
COc1cc(C#N)cc(C(=O)Nc2cnc3c(c(C4CCN(C(=O)C(C)C)CC4)cn3C)c2C(F)(F)F)c1
CC(=O)N1CCN(c2ccc(CN(C3CCC3)S(=O)(=O)Cc3ccccc3C(F)(F)F)c(F)c2)CC1
CC(=O)N1CCC(Nc2ccc(CN(CC(C)C)S(=O)(=O)Cc3ccccc3)cc2)CC1
CC(=O)N1CCN(c2ccc(CN(CC(C)C)S(=O)(=O)Cc3ccccc3)cc2)C(=O)C1
CC(C)CN(Cc1ccc(N2CC(C(N)=O)C2)cc1)S(=O)(=O)Cc1ccccc1
CC(C)CN(Cc1ccc(N2CCC3(COC3)C2)cc1)S(=O)(=O)Cc1ccccc1
CC(C)CN(Cc1ccc(F)cc1)S(=O)(=O)N(C)c1ccc(OC2CCN(S(C)(=O)=O)CC2)cc1
CC(=O)N1CCN(c2ccc(CN(C(C)C)S(=O)(=O)Cc3ccccc3)cc2)CC1
CC(C)CN(Cc1ccc(N2CCN(C)CC2)cc1)S(=O)(=O)Cc1ccccc1
Cc1c(NC(=O)c2cccc(C#N)c2)cnc2c1c(C1CC3CCC1CN3C(=O)C1CCCC1)cn2C
CC(=O)N1CCN(c2ccc(CN(CC(C)C)S(=O)(=O)Cc3ccccc3)c(F)c2)CC1
CC(C)CN(Cc1cccc(Cl)c1)S(=O)(=O)c1ccc(-c2ccc(S(C)(=O)=O)cc2)cc1
Cc1c(NC(=O)c2cccc(C#N)c2)cnc2c1c(C1CC(C)N(C(=O)C3CCCC3)CC1C)cn2C
CC(=O)N1CCN(c2ccc(CN(CC(C)C)S(=O)(=O)Cc3ccccc3)cc2C#N)CC1
CC(=O)N1CCN(c2ccc(CN(C3CCC3)S(=O)(=O)Oc3ccccc3)c(F)c2)CC1
CCCN(Cc1ccc(N2CCN(C(C)=O)CC2)cc1)S(=O)(=O)Cc1ccccc1
COc1ccc(C(=O)Nc2cnc3c(c(C4CCN(C(=O)C(C)C(C)(C)C)CC4)cn3C)c2C)cc1C#N
Cc1c(NC(=O)c2cc(O)cc(C#N)c2)cnc2c1c(C1CCN(C(=O)C3CCCC3)CC1)cn2C
Cn1cc(C2CCN(C(=O)CC3CCCC3)CC2)c2c(C(F)(F)F)c(NC(=O)c3cccc(C#N)c3)cnc21
CC(=O)N1CCC2(C1)CN(c1ccc(CN(CC(C)C)S(=O)(=O)Cc3ccccc3)cc1)C2
COc1cc(C#N)cc(C(=O)Nc2cnc3c(c(C4CCN(C(=O)C(C)C(C)(C)C)CC4)cn3C)c2C)c1

```
COc1ccc(C(=O)Nc2cnc3c(c(C4CCN(C(=O)C(C)C(C)(C)C)C(C)(C)C4)cn3C)c2C)cc1C#N
Cn1cc(C2C3CCC2CN(C(=O)C2CCCC2)C3)c2c(C(F)(F)F)c(NC(=O)c3cccc(C#N)c3)cnc21
CNC(=O)C1CCN(c2ccc(CN(CC(C)C)S(=O)(=O)Cc3ccccc3)cc2)CC1
CC(=O)Nc1ccc2c(c1)CCCN2Cc1ccc(C(O)(C(F)(F)F)C(F)(F)F)cc1
Cc1c(NC(=O)c2cccc(C#N)c2)cnc2c1c(C1CCN(C(=O)C(C)C3CC3)CC1C)cn2C
COC(C)C(=O)N1CCN(c2ccc(CN(CC(C)C)S(=O)(=O)Cc3ccccc3)cc2)CC1
CC(=O)N1CCN(c2ccc(CN(C3CCC3)S(=O)(=O)c3cccc(C#N)c3)c(F)c2)CC1
CC(=O)N1CCN(c2ccc(CN(C3CCC3)S(=O)(=O)c3ccc(Cl)cc3Cl)c(F)c2)CC1
CC(=O)N1CC2CC1CN2c1ccc(CN(CC(F)(F)F)S(=O)(=O)Cc2ccccc2)c(F)c1
CC(C)CN(C(C)c1ccc(N2CCC(C(N)=O)CC2)cc1)S(=O)(=O)Cc1ccccc1
Cc1c(NC(=O)c2cccc(C#N)c2)cnc2c1c(C1CCN(C(=O)C3CCCC3)C(C)(C)C1)cn2C
Cn1cc(C2CCN(C(=O)C3CCCS3)CC2)c2c(C(F)(F)F)c(NC(=O)c3cccc(C#N)c3)cnc21
CC(=O)N1CCN(c2ccc(CN(C3CCC3)S(=O)(=O)c3cccc(F)c3)c(F)c2)CC1
CC(=O)N1CCN(c2ccc(CN(C3CCC3)S(=O)(=O)c3ccccc3C(F)(F)F)c(F)c2)CC1
CC(C)CN(Cc1ccc(N2CCN(C(=O)C3COC3)CC2)cc1)S(=O)(=O)Cc1ccccc1
CC(C)CN(Cc1ccc(N2CCN(C3COC3)CC2)cc1)S(=O)(=O)Cc1ccccc1
CC(=O)N1CCN(c2ccc(CN(C3CCC3)S(=O)(=O)Cc3ccc(Cl)c(Cl)c3)c(F)c2)CC1
CC(=O)N1CCN(c2ccc(CN(C3CCC3)S(=O)(=O)c3ccc(C(C)(C)C)cc3)c(F)c2)CC1
CC(C)CN(Cc1ccc(N2CCC(N(C)C)C2)cc1)S(=O)(=O)Cc1ccccc1
CC(=O)N1CCN(c2ccc(CN(C3CCC3)S(=O)(=O)c3cccc(Cl)c3)c(F)c2)CC1
CC(C)CN(c1cccc(Cl)c1)S(=O)(=O)c1ccc(-c2ccc(NS(C)(=O)=O)cc2)cc1
Cc1c(NC(=O)c2cccc(C#N)c2)cnc2c1c(C1CCN(C(=O)C(C)C)C(C)(C)C1)cn2C
CC(=O)N1CCN(c2ccc(CN(C3(C)CC3)S(=O)(=O)Cc3ccccc3)cc2)CC1
CC(C)CN(Cc1ccc(N2CCN(S(C)(=O)=O)CC2)cc1)S(=O)(=O)Cc1ccccc1
CC(C)C(=O)N1CCC(c2cn(C)c3ncc(NC(=O)c4cccc(C#N)c4)c(C(F)(F)F)c23)CC1C
Cc1c(NC(=O)c2cccc(C#N)c2)cnc2c1c(C1C3CCC1CN(C(=O)C(C)C)C3)cn2C
CC(C)CN(c1ccccc1Cl)S(=O)(=O)c1cccc(-c2ccc(S(C)(=O)=O)cc2)c1
CC(C)C(=O)N1CC2CCC(C1)C2c1cn(C)c2ncc(NC(=O)c3cccc(C#N)c3)c(C(F)(F)F)c12
COC(=O)N1CCN(c2ccc(CN(CC(C)C)S(=O)(=O)Cc3ccccc3)cc2)CC1
Cc1c(NC(=O)c2cccc(C#N)c2)cnc2c1c(C1CCN(C(=O)C3CCCC3)CC1C)cn2C
CS(=O)(=O)Nc1ccc2c(c1)CCC(=O)N2Cc1ccc(C(O)(C(F)(F)F)C(F)(F)F)cc1
Cc1c(NC(=O)c2cccc(C#N)c2)cnc2c1c(C1CCN(C(=O)C(C)C(C)(C)C)CC1)cn2C
CC(C)CN(Cc1ccc(N2CCC(N(C)C)CC2)cc1)S(=O)(=O)Cc1ccccc1
Cc1ccc(C(=O)Nc2cnc3c(c(C4CCN(C(=O)C5CCCC5)C(C)(C)C4)cn3C)c2C)cc1C#N
CC(=O)N1CCN(c2ccc(CN(C3CCC3)S(=O)(=O)c3cccc(Cl)c3Cl)c(F)c2)CC1
COc1ccc(C(=O)Nc2cnc3c(c(C4CCN(C(=O)C(C)C(F)(F)F)CC4)cn3C)c2C)cc1C#N
CC(C)CN(Cc1ccc(NC2(C)CCN(S(C)(=O)=O)CC2)cc1)S(=O)(=O)Cc1ccccc1
COc1ccc(C(=O)Nc2cnc3c(c(C4CCN(C(=O)C5CCCC5)C(C)(C)C4)cn3C)c2C)cc1C#N
CS(=O)(=O)NC1CCN(c2ccc(CN(CC(F)(F)F)S(=O)(=O)Cc3ccccc3)c(F)c2)CC1
CC(C)CN(Cc1ccccc1)S(=O)(=O)c1ccc(-c2ccc(C(N)=O)cc2)cc1
CC(=O)N1CCN(c2ccc3c(c2)OCCN(S(=O)(=O)Cc2ccccc2)C3)CC1
CC(C)CN(Cc1ccc(N2CCCC(O)C2)cc1)S(=O)(=O)Cc1ccccc1
Cc1c(NC(=O)c2cccc(C#N)c2)cnc2c1c(C1CCN(C(=O)C(C)C(C)(C)C)C(C)(C)C1)cn2C
CC(C)CN(Cc1ccc(N2CCC(CO)CC2)cc1)S(=O)(=O)Cc1ccccc1
CC(=O)N1CC2(CCN(c3ccc(CN(CC(C)C)S(=O)(=O)Cc4ccccc4)cc3)C2)C1
CC(C)CN(Cc1ccc(N2CCC3(CCCO3)C2)cc1)S(=O)(=O)Cc1ccccc1
CCN1CCC(Nc2ccc(CN(CC(C)C)S(=O)(=O)Cc3ccccc3)cc2)C(F)C1
CC(C)C(=O)N1CCC(c2cn(C)c3ncc(NC(=O)c4ccc(F)c(Cl)c4)c(C(F)(F)F)c23)CC1
CC(C)CN(Cc1ccc(N2CC3(CCOCC3)C2)cc1)S(=O)(=O)Cc1ccccc1
Cn1cc(C2CCN(C(=O)C3CC4CCCCC4C3)CC2)c2c(C(F)(F)F)c(NC(=O)c3cccc(C#N)c3)cnc21
CC(=O)N1CCN(c2ccc(CN(CC(C)C)S(=O)(=O)Cc3ccccc3)c(C)c2)CC1
CC(C)CN(C(C)c1ccc(N2CCN(C)CC2)cc1)S(=O)(=O)Cc1ccccc1
Cc1c(NC(=O)c2cccc(C#N)c2)cnc2c1c(C1CCN(C(=O)C(C)C3CCCC3)CC1)cn2C
CC(=O)N1C2CC1CN(c1ccc(CN(CC(F)(F)F)S(=O)(=O)Cc3ccccc3)c(F)c1)C2
CC(C)CN(C(C)c1ccc(N2CCOCC2)cc1)S(=O)(=O)Cc1ccccc1
CC(=O)N1CCN(c2ccc(CN(CC(F)(F)F)S(=O)(=O)Cc3ccccc3)cc2)CC1
CC(=O)N1CCN(c2ccc(CN(C3CCC3)S(=O)(=O)Cc3cc(Cl)cc(Cl)c3)c(F)c2)CC1
```

CC(=O)N1CCN(c2ccc(CN(C3COC3)S(=O)(=O)Cc3ccccc3)cc2)CC1
CC(=O)N1CCN(c2ccc(CN(C3CCC3)S(=O)(=O)Cc3ccc(F)cc3)c(F)c2)CC1
CC(C)CN(Cc1ccc(NC2CCCC(O)C2)cc1)S(=O)(=O)Cc1ccccc1
CC(C)CN(Cc1ccccc1)S(=O)(=O)c1ccc(-c2ccc(S(C)(=O)=O)cc2)cc1
COc1ccc(S(=O)(=O)N(Cc2ccc(N3CCN(C(C)=O)CC3)cc2F)C2CCC2)c(OC)c1
CC(=O)N1CCN(c2ccc(CN(C3CCC3)S(=O)(=O)Cc3ccc(C(F)(F)F)cc3)c(F)c2)CC1
Cc1c(NC(=O)c2cccc(C#N)c2)cnc2c1c(C1CCN(C(=O)c3cccc4c3CCC4)CC1)cn2C
CC(C)CN(c1ccc(Cl)cc1)S(=O)(=O)c1ccc(-c2ccc(S(C)(=O)=O)cc2)cc1
CC(=O)N1CCN(c2ccc(CN(CC#N)S(=O)(=O)Cc3ccccc3)cc2)CC1
CC(C)CN(Cc1ccc(N2CCC(C#N)CC2)cc1)S(=O)(=O)Cc1ccccc1
Cn1cc(C2CCN(C(=O)CC3CCCCC3)CC2)c2c(C(F)(F)F)c(NC(=O)c3cccc(C#N)c3)cnc21
CC(C)C(=O)N1CCC(c2cn(C)c3ncc(NC(=O)c4cccc(C#N)c4F)c(C(F)(F)F)c23)C(C)C1
CC(C)CN(Cc1ccc(F)cc1)S(=O)(=O)Nc1ccc(OC2CCN(S(C)(=O)=O)CC2)cc1
CC(=O)N1CCN(c2ccc(CN(C3CCC3)S(=O)(=O)Cc3ccccc3Cl)c(F)c2)CC1
CC(=O)N1CCN(c2ccc(CN(C3CCC3)S(=O)(=O)c3cc(C)cc(C)c3)c(F)c2)CC1
CS(=O)(=O)N(Cc1ccc(N2CCN(C(=O)C3CC3)CC2)cc1F)S(=O)(=O)CCc1ccccc1
CC(C)CN(Cc1ccc(N2CCC3(CCOC3)C2)cc1)S(=O)(=O)Cc1ccccc1
Cc1c(NC(=O)c2cccc(C#N)c2)cnc2c1c(C1CCN(C(=O)CC(C)C)CC1)cn2C
CC(C)CN(c1cccc(Cl)c1)S(=O)(=O)c1ccc(-c2ccc(S(N)(=O)=O)cc2)cc1
Cc1ccc(C(=O)Nc2cnc3c(c(C4CCN(C(=O)C5CCCC5)CC4)cn3C)c2C(F)(F)F)cc1C#N
COc1ccc(C(=O)Nc2cnc3c(c(C4CCN(C(=O)C(C)C)CC4)cn3C)c2C(F)(F)F)cc1Cl
CC(C)CN(Cc1ccc(NC2CCCOC2)cc1)S(=O)(=O)Cc1ccccc1
COCCNc1ccc(CN(CC(C)C)S(=O)(=O)Cc2ccccc2)cc1
COc1ccc(C(=O)Nc2cnc3c(c(C4CCN(C(=O)C(C)C)C(C)(C)C4)cn3C)c2C)cc1C#N
Cn1cc(C2CCN(C(=O)C3CCCC3)CC2)c2c(C(F)(F)F)c(NC(=O)c3cccc(C#N)c3)cnc21
CC(C)CN(Cc1ccc(N2CCN(C(=O)CO)CC2)cc1)S(=O)(=O)Cc1ccccc1
CC(=O)N1CCN(c2ccc(CN(C(C)(C)C)S(=O)(=O)Cc3ccccc3)cc2)CC1
CC(=O)Nc1ccc2c(c1)CCN2Cc1ccc(C(O)(C(F)(F)F)C(F)(F)F)cc1
CC(C)C(=O)N1CCC(c2cn(C)c3ncc(NC(=O)c4cccc(C#N)c4)c(C(F)(F)F)c23)CC1(C)C
CC(C)CN(Cc1ccc(N2CCC(S(C)(=O)=O)C2)cc1)S(=O)(=O)Cc1ccccc1
CC(=O)N1CCN(c2ccc(CN3CCC(C)N(c4ccccc4)S3(=O)=O)c(F)c2)CC1

Table S2 | Comparison of the fraction of sp^3-hybridized carbon atoms (Fsp^3) between a dataset with synthetic molecules (ChEMBL), a dataset of natural products (MEGx) and the de novo designs **1**, **2** and **3**. The mean ± standard deviation is reported.

Data	**Fsp^3**
ChEMBL	0.33 ± 0.2
MEGx	0.51 ± 0.3
Design **1**	0.65
Design **2**	0.50
Design **3**	0.50

Table S3 | Loadings for the first two principal components (PCs) depicted in Figure S3.

**Descriptors**	**PC 1**	**PC 2**
Fraction of sp^3-hybridized carbon atoms	0.08	0.70
Polar surface area	0.57	-0.10
Number of aliphatic rings	0.05	0.71
Number of hydrogen bond acceptors	0.55	-0.05
Number of nitrogens and oxygens	0.60	-0.02

Analytical data (NMR, HRMS, HPLC)

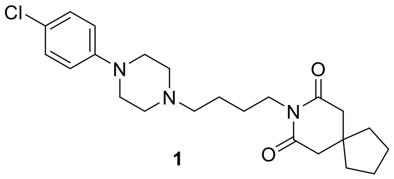

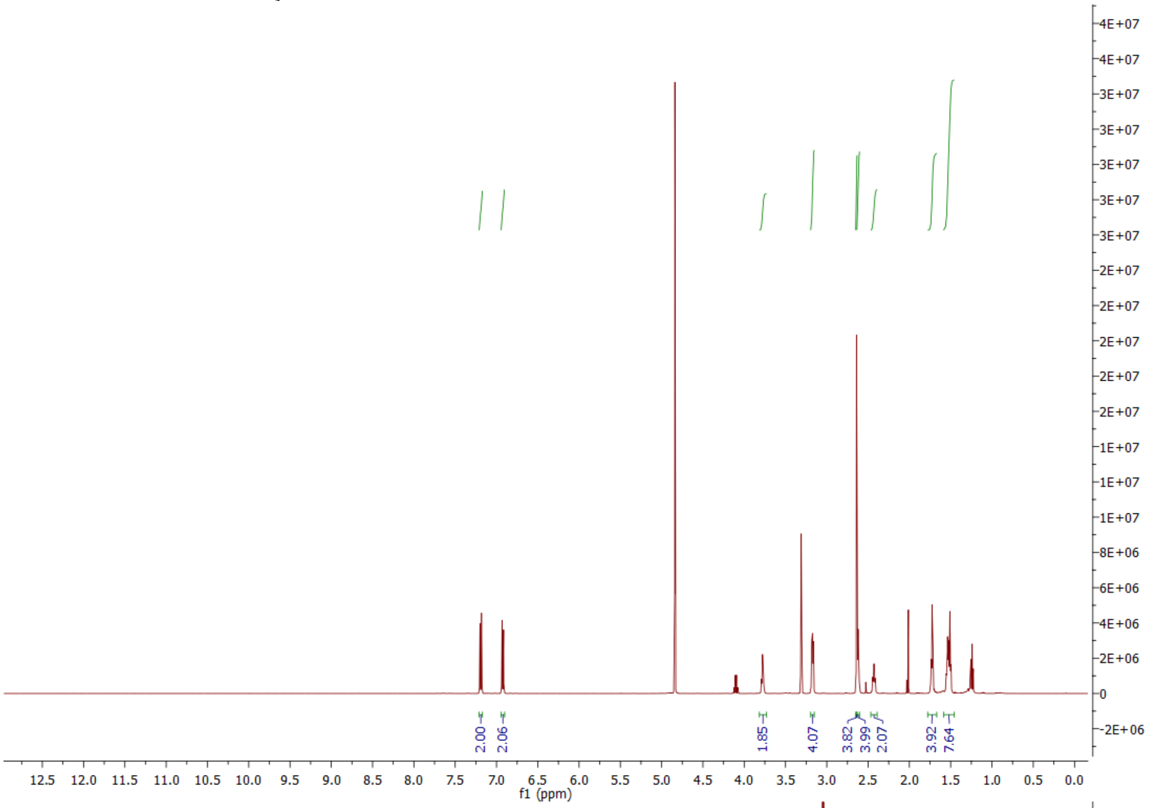

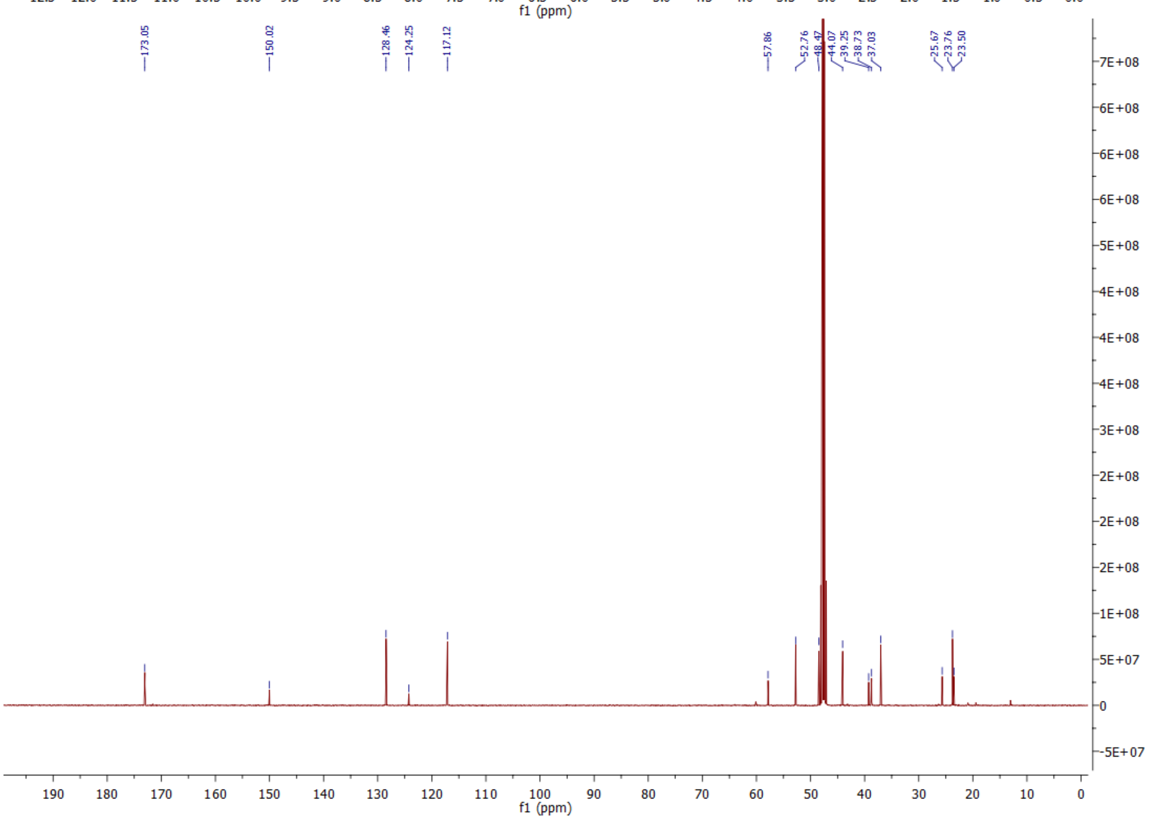

Chrom Type: Fixed WL Chromatogram, 254 nm

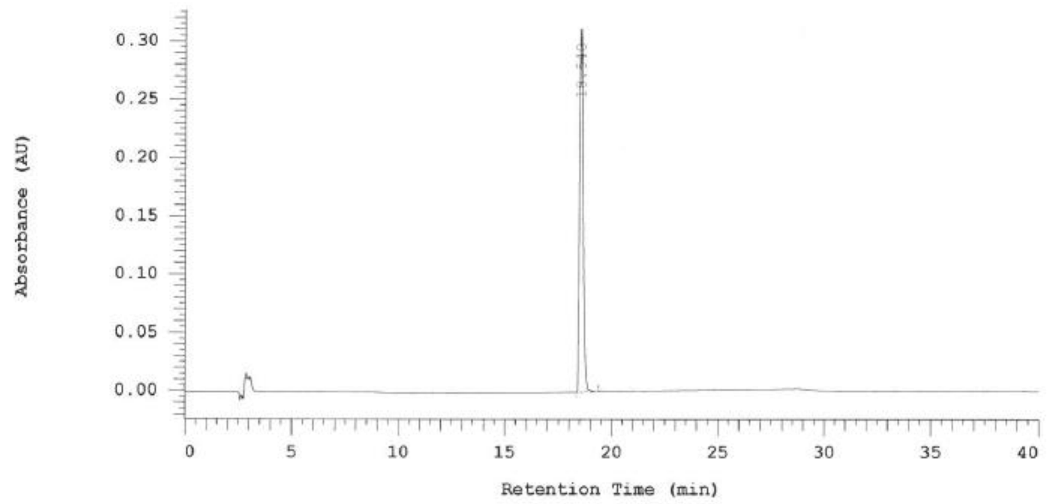

Pump 1: 5160
Pump 1 Solvent A: MeOH
Pump 1 Solvent B: Wasser
Pump 1 Solvent C: MeOH
Pump 1 Solvent D: MeOH

No.	RT	Area	Area %	Height
1	18.540	1676145	100.000	155660
		1676145	100.000	155660

Chrom Type: Fixed WL Chromatogram, 280 nm

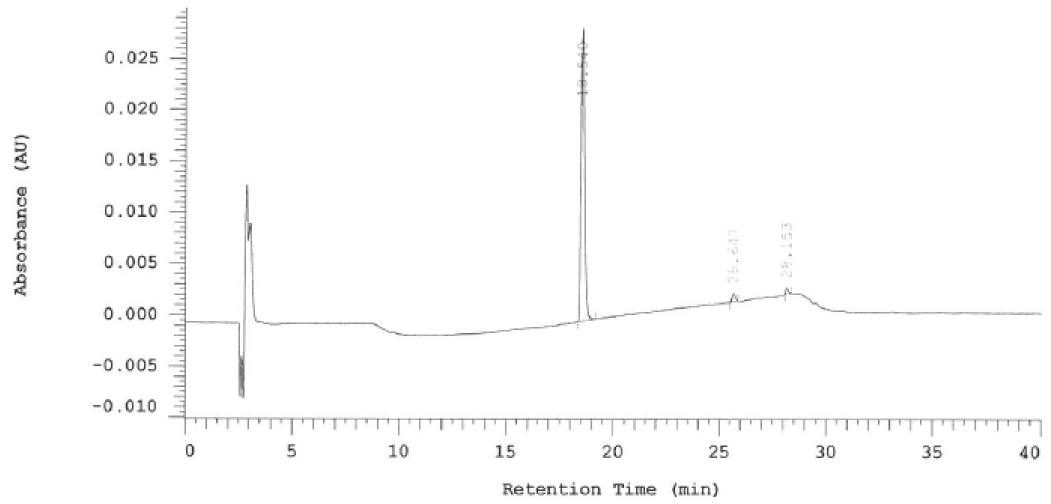

Pump 1: 5160
Pump 1 Solvent A: MeOH
Pump 1 Solvent B: Wasser
Pump 1 Solvent C: MeOH
Pump 1 Solvent D: MeOH

No.	RT	Area	Area %	Height
1	18.540	155005	95.995	14302
2	25.647	3984	2.468	372
3	28.153	2481	1.537	280
		161470	100.000	14954

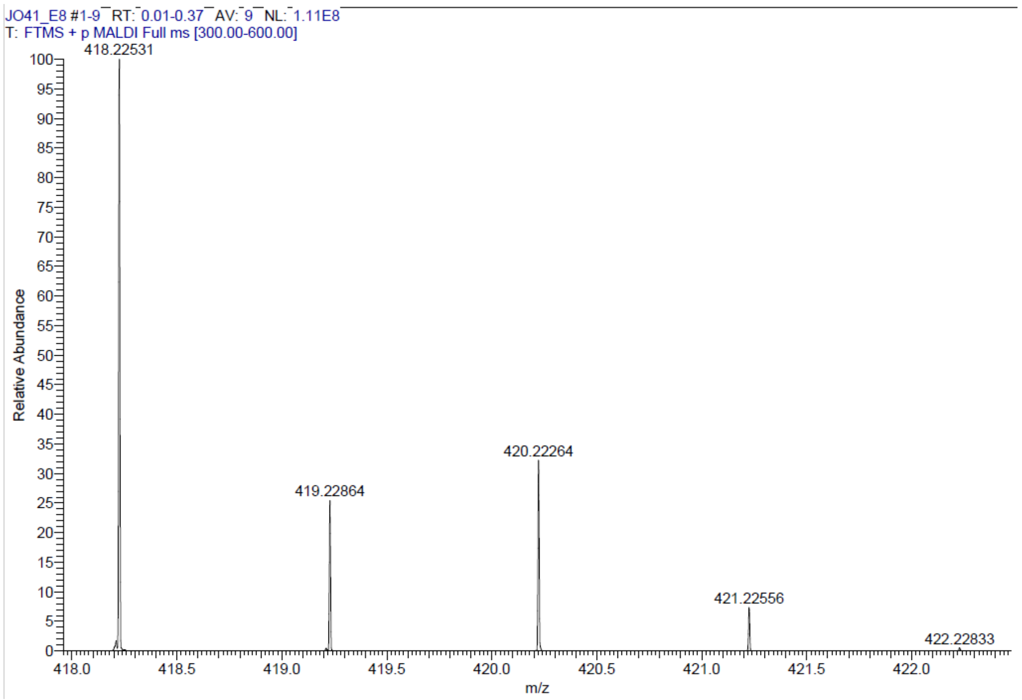

C23H32Cl1N3O2 +H: C23 H33 Cl1 N3 O2 pa Chrg 1

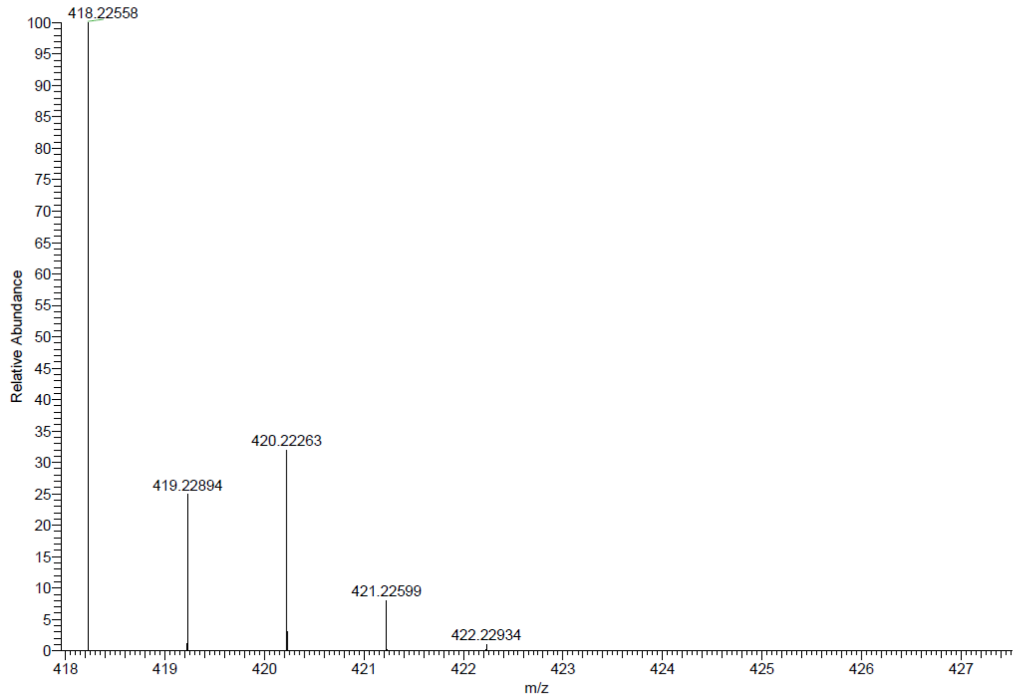

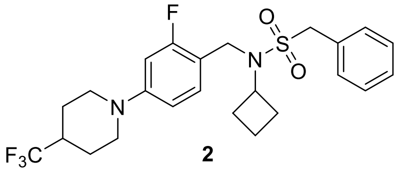
F
O
S
O
N
N
F3C

2

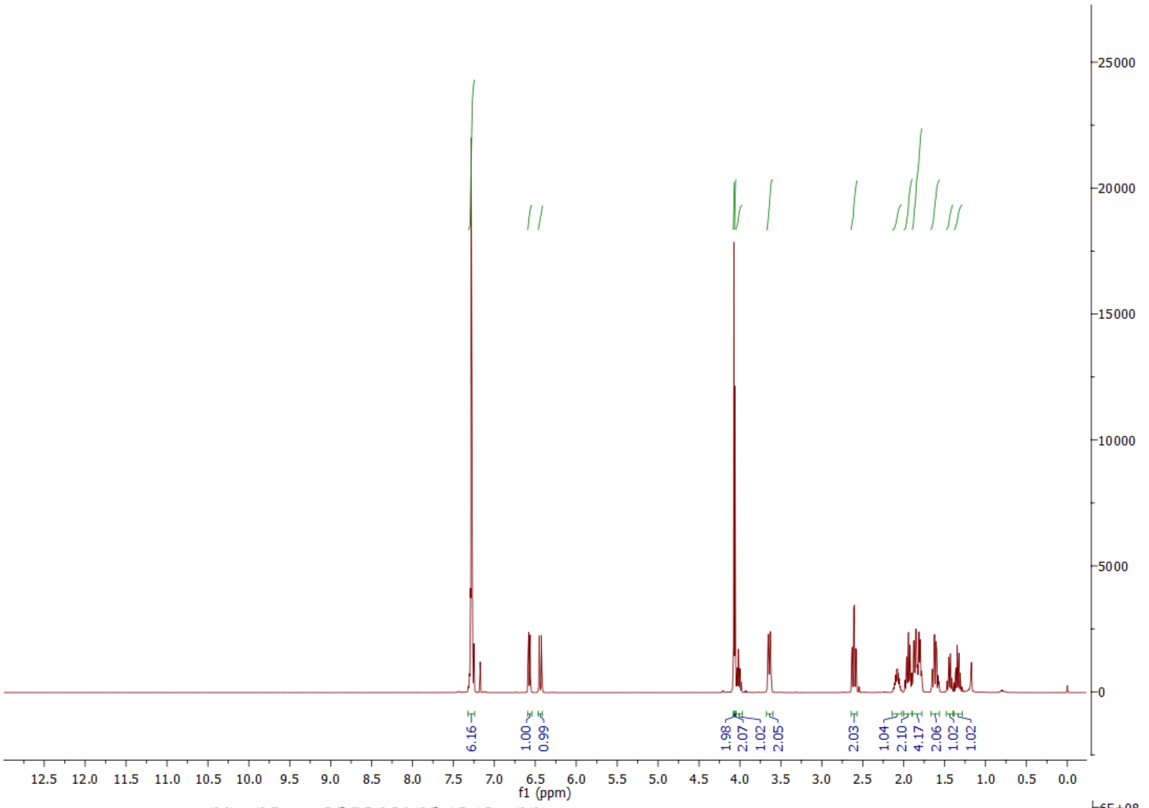
6.16
1.00
0.99
1.98
2.07
1.02
2.05
2.03
1.04
2.10
4.17
2.06
1.02
1.02
f1 (ppm)

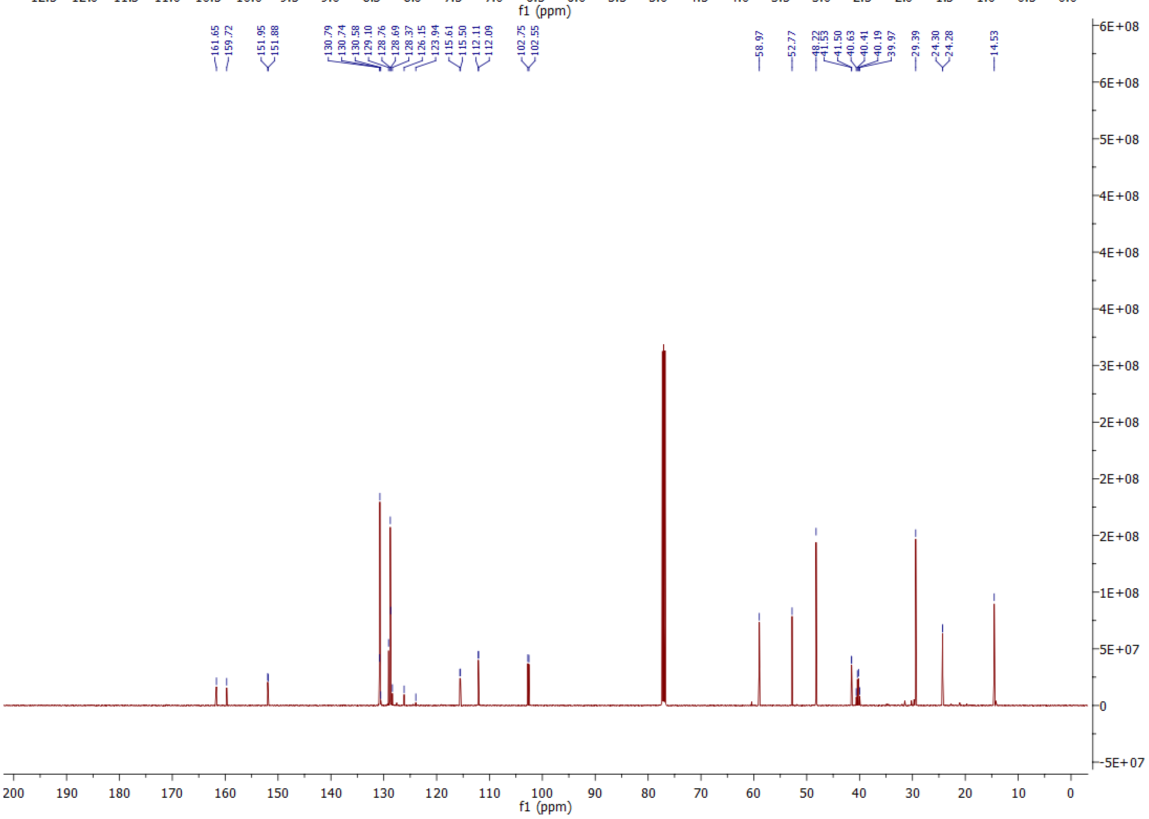
161.65
159.72
151.95
151.88
130.79
130.74
130.58
129.10
128.76
128.69
128.37
126.15
123.94
115.61
115.50
112.11
112.09
102.75
102.55
58.97
52.77
48.22
41.53
41.50
40.63
40.41
40.19
39.97
29.39
24.30
24.28
14.53
f1 (ppm)

Chrom Type: Fixed WL Chromatogram, 254 nm

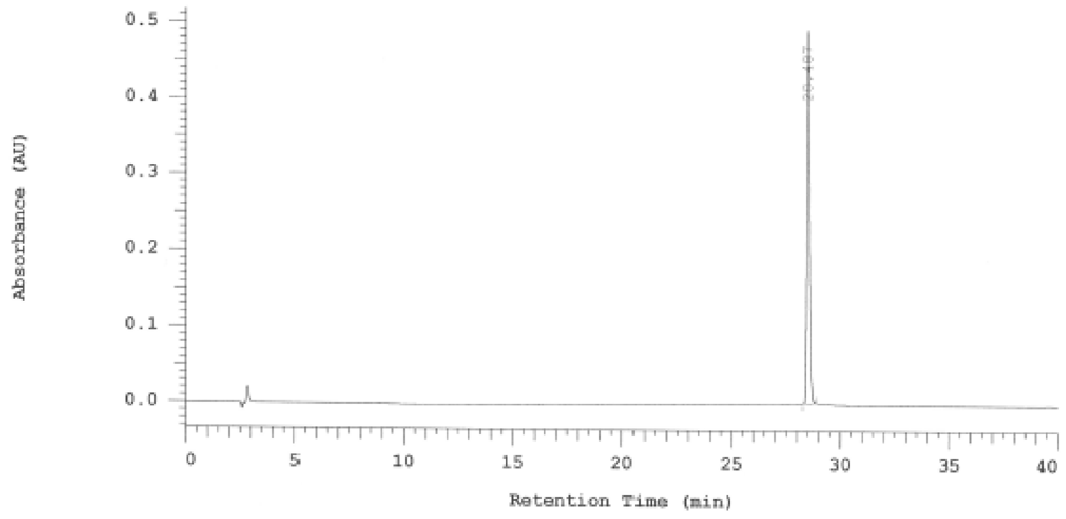

Pump 1: 5160
Pump 1 Solvent A: MeOH
Pump 1 Solvent C: MeOH
Pump 1 Solvent B: Wasser
Pump 1 Solvent D: MeOH

No.	RT	Area	Area %	Height
1	28.487	2148567	100.000	244683
		2148567	100.000	244683

Chrom Type: Fixed WL Chromatogram, 280 nm

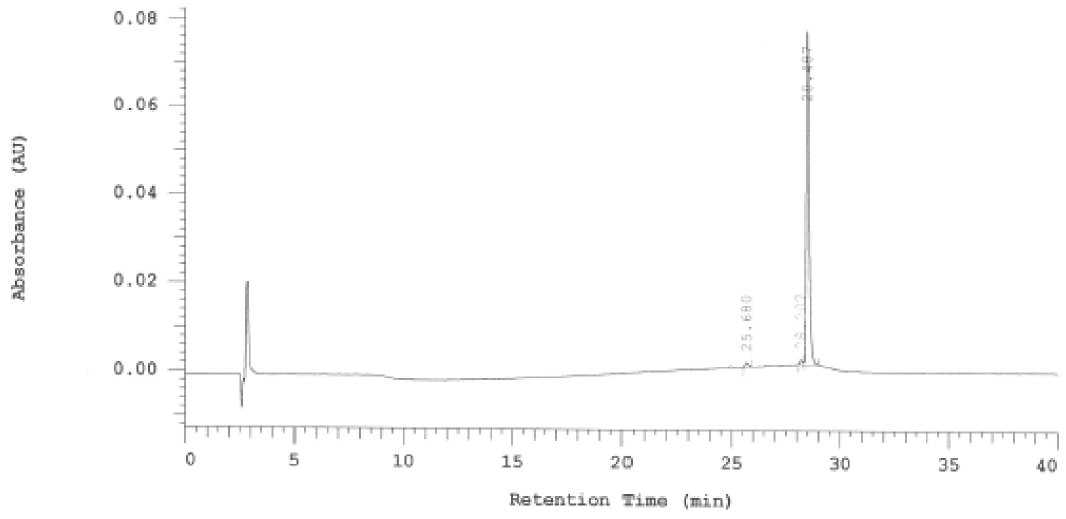

Pump 1: 5160
Pump 1 Solvent A: MeOH
Pump 1 Solvent C: MeOH
Pump 1 Solvent B: Wasser
Pump 1 Solvent D: MeOH

No.	RT	Area	Area %	Height
1	25.680	5682	1.629	542
2	28.207	4467	1.281	474
3	28.487	338596	97.090	37870
		348745	100.000	38886

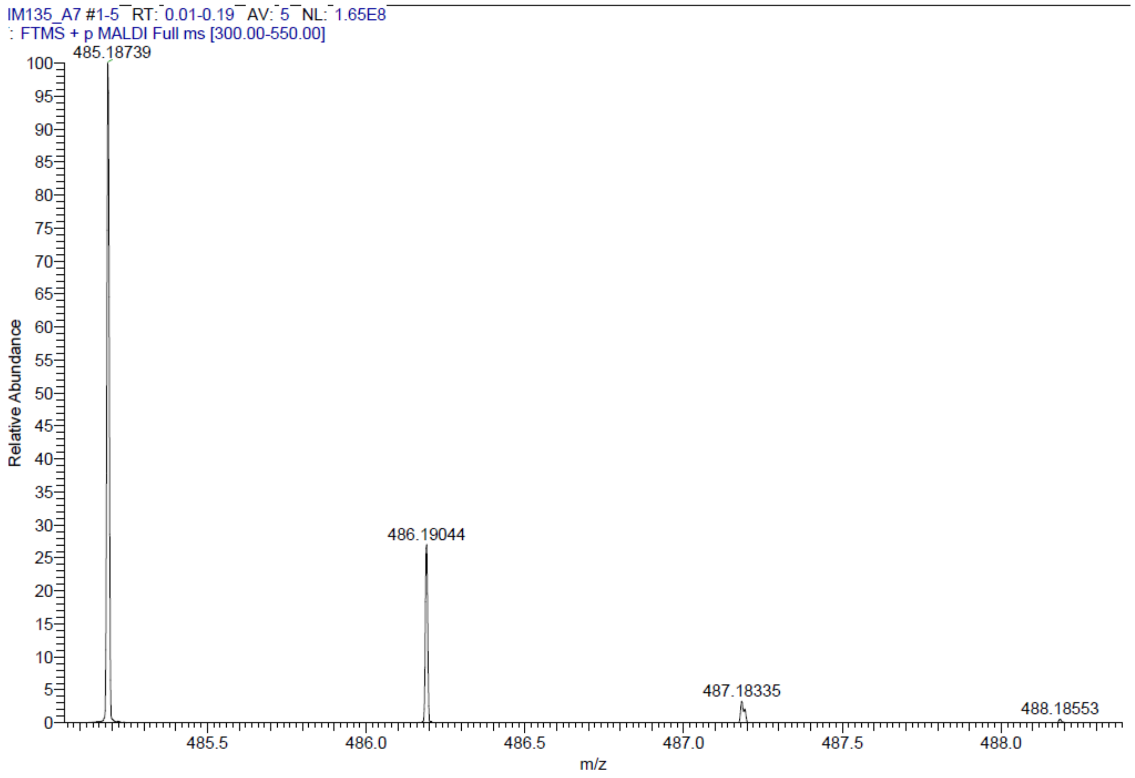
IM135_A7 #1-5 RT: 0.01-0.19 AV: 5 NL: 1.65E8
: FTMS + p MALDI Full ms [300.00-550.00]
485.18739
486.19044
487.18335
488.18553
Relative Abundance
m/z

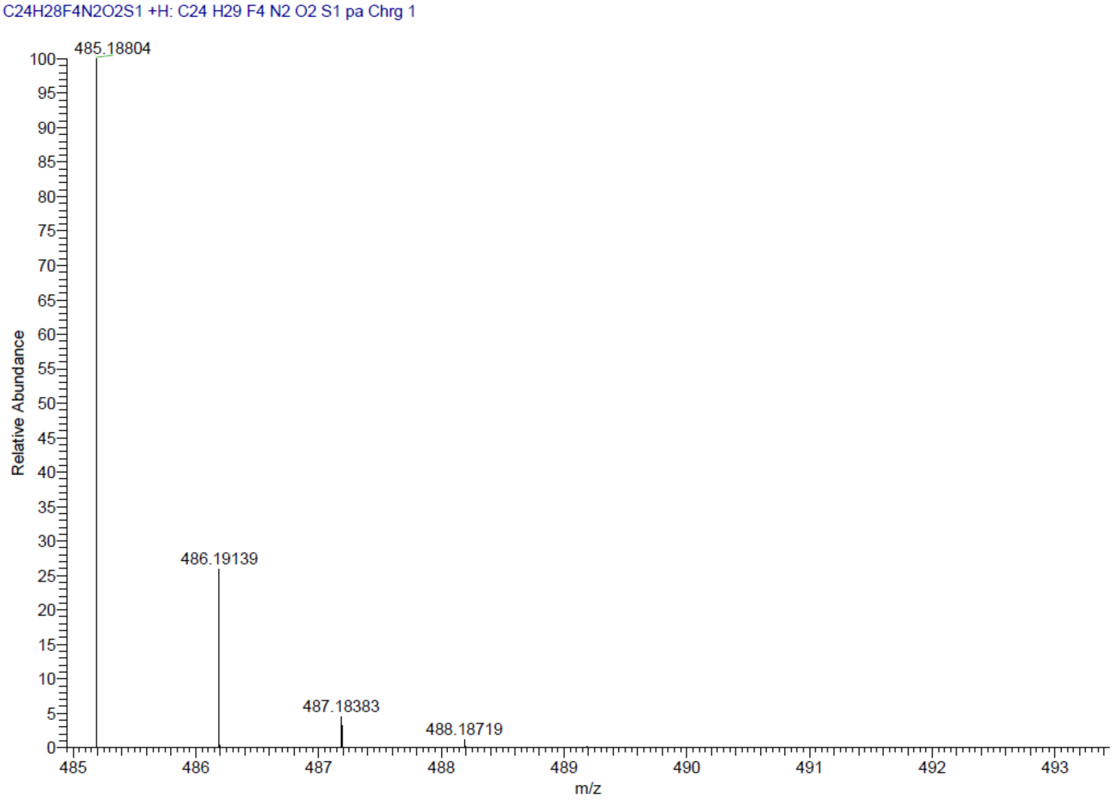
C24H28F4N2O2S1 +H: C24 H29 F4 N2 O2 S1 pa Chrg 1
485.18804
486.19139
487.18383
488.18719
Relative Abundance
m/z

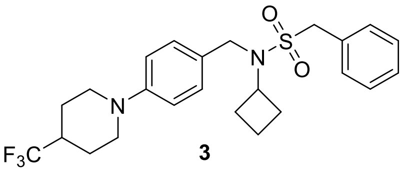
O
S
O
N
N
F3C

3

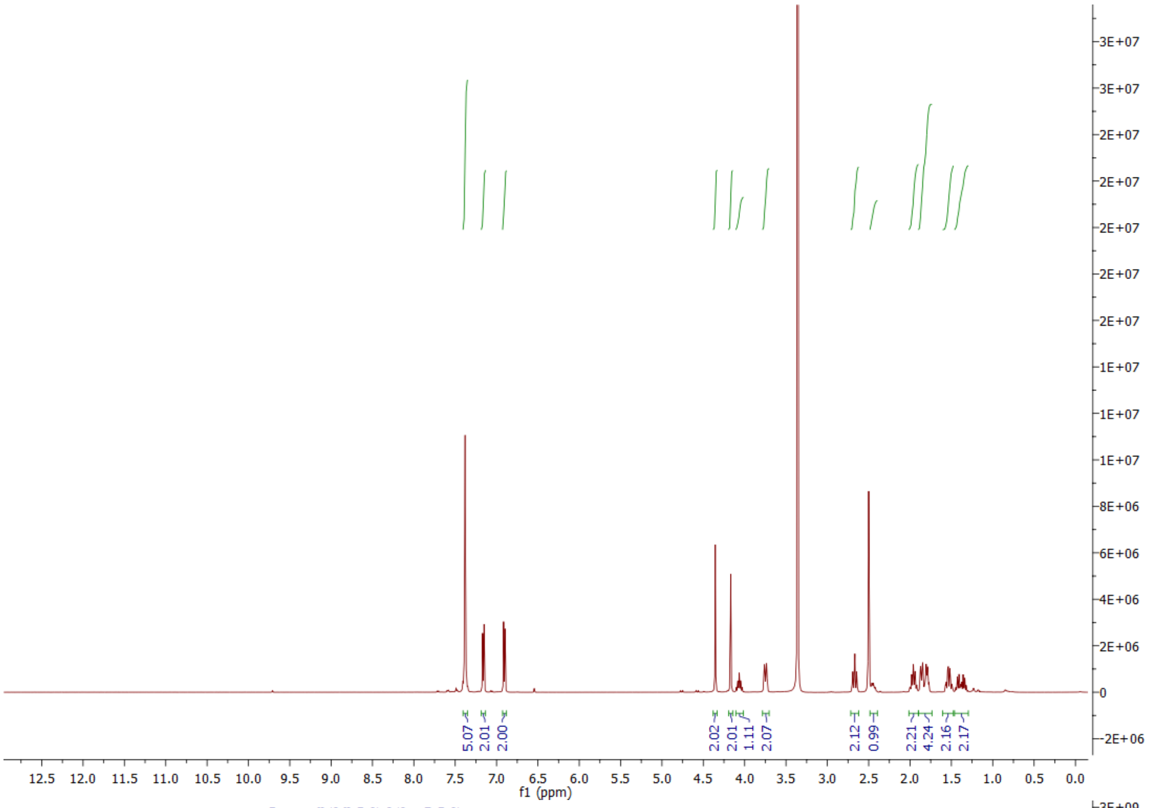
5.07
2.01
2.00
2.02
2.01
1.11
2.07
2.12
0.99
2.21
4.24
2.16
2.17
f1 (ppm)

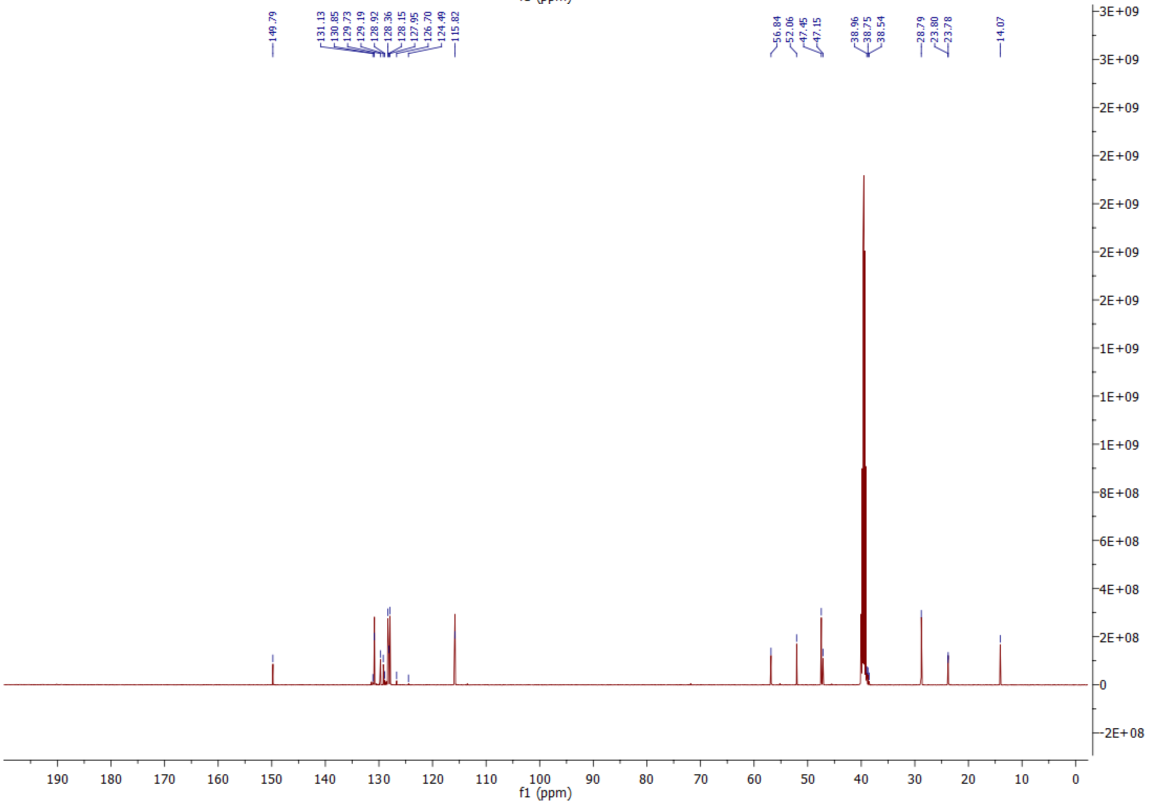
149.79
131.13
130.85
129.73
129.19
128.92
128.36
128.15
127.95
126.70
124.49
115.82
56.84
52.06
47.45
47.15
38.96
38.75
38.54
28.79
23.80
23.78
14.07
f1 (ppm)

Chrom Type: Fixed WL Chromatogram, 254 nm

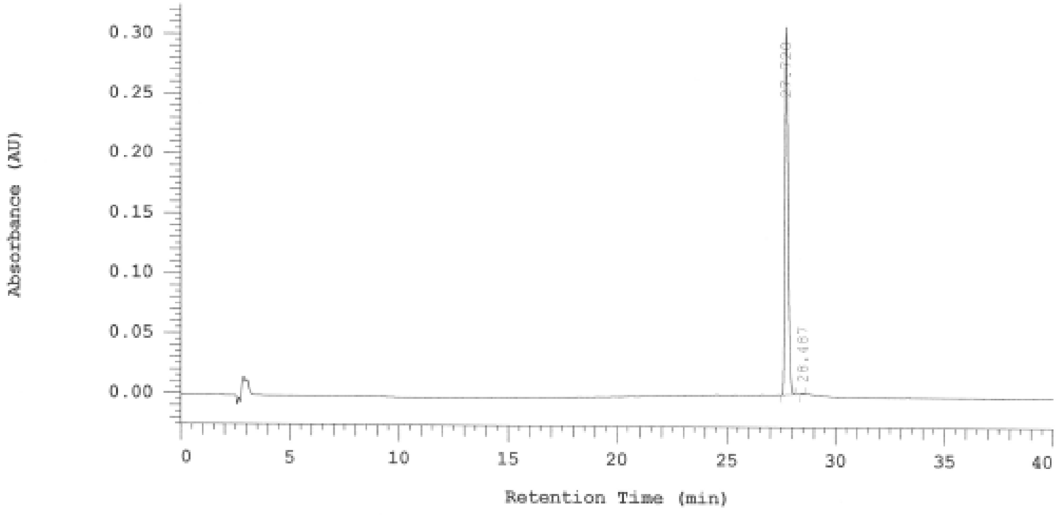

Pump 1: 5160
Pump 1 Solvent A: MeOH
Pump 1 Solvent C: MeOH
Pump 1 Solvent B: Wasser
Pump 1 Solvent D: MeOH

No.	RT	Area	Area %	Height
1	27.720	1414706	99.749	153130
2	28.487	3556	0.251	448
		1418262	100.000	153578

Chrom Type: Fixed WL Chromatogram, 254 nm

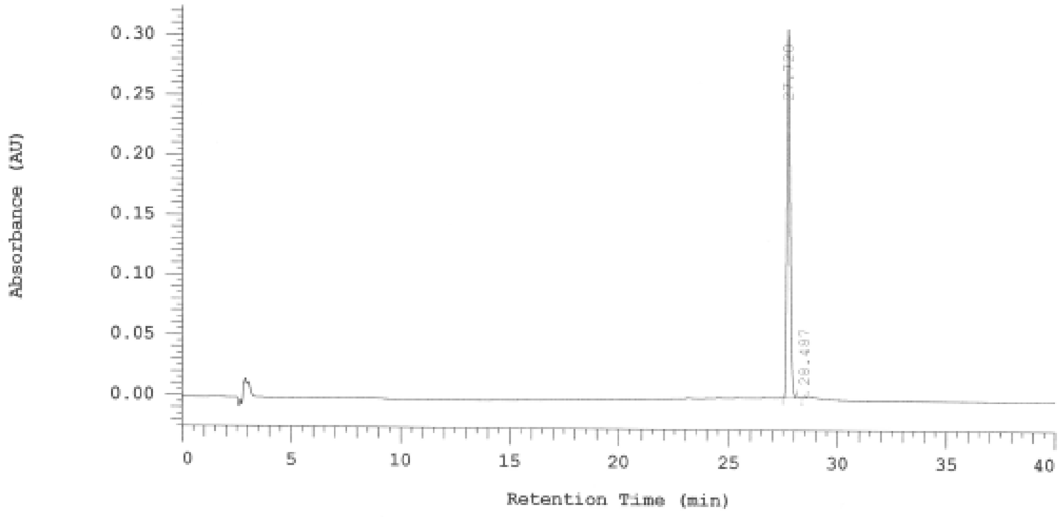

Pump 1: 5160
Pump 1 Solvent A: MeOH
Pump 1 Solvent C: MeOH
Pump 1 Solvent B: Wasser
Pump 1 Solvent D: MeOH

No.	RT	Area	Area %	Height
1	27.720	1414706	99.749	153130
2	28.487	3556	0.251	448
		1418262	100.000	153578

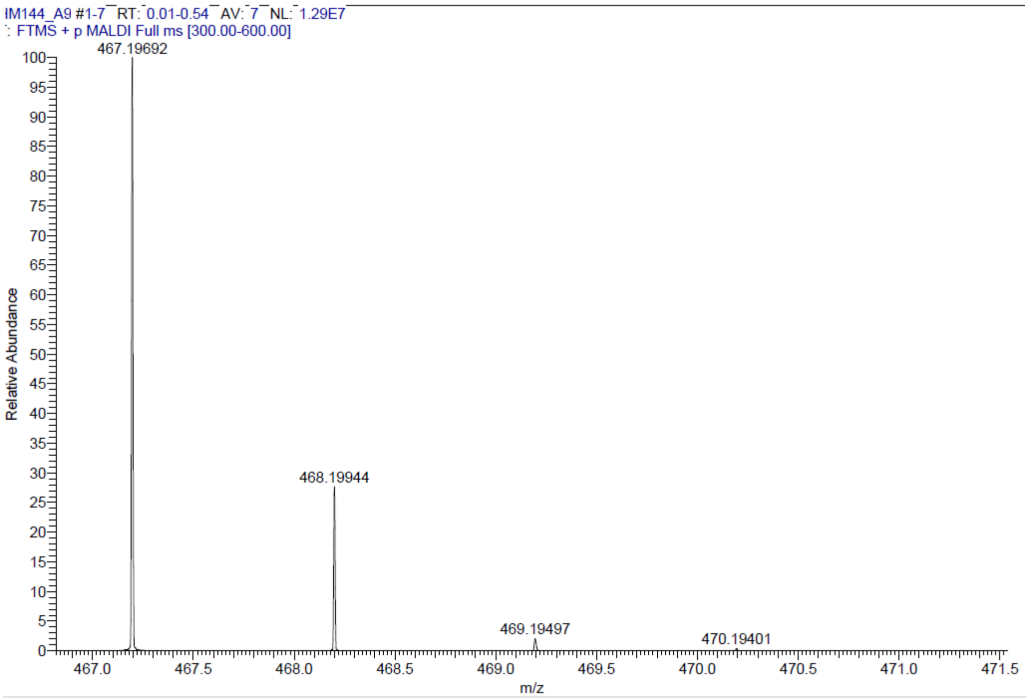
IM144_A9 #1-7 RT: 0.01-0.54 AV: 7 NL: 1.29E7
: FTMS + p MALDI Full ms [300.00-600.00]
467.19692
468.19944
469.19497
470.19401
Relative Abundance
m/z

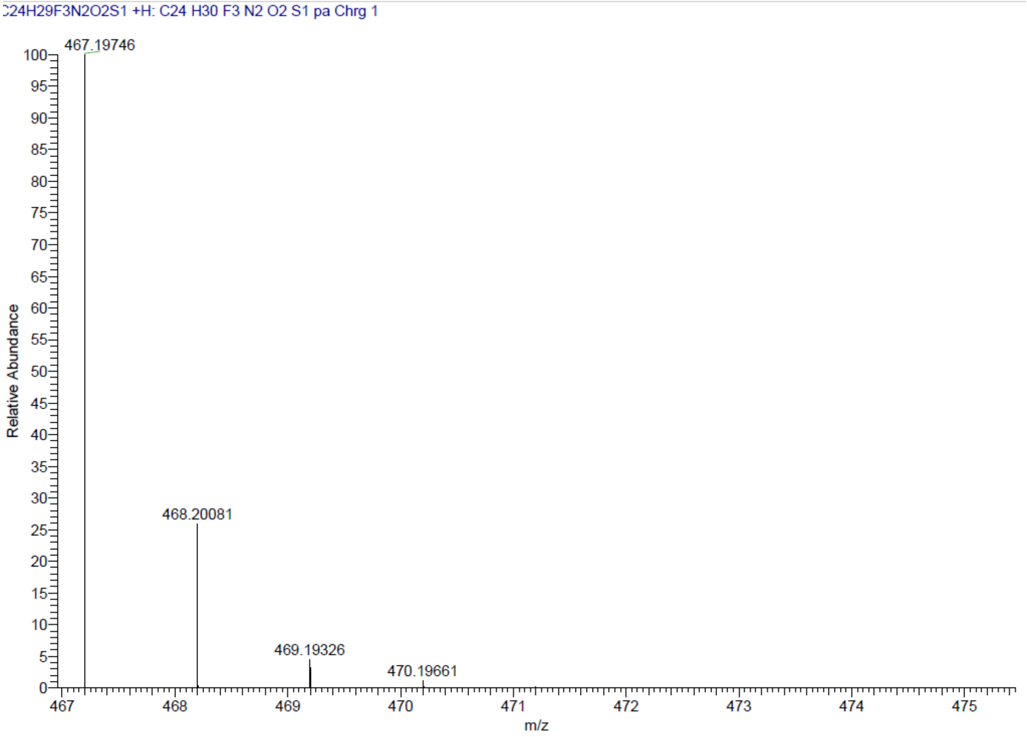
C24H29F3N2O2S1 +H: C24 H30 F3 N2 O2 S1 pa Chrg 1
467.19746
468.20081
469.19326
470.19661
Relative Abundance
m/z

References

[1] D. Weininger, *J. Chem. Inf. Comput. Sci.* **1988**, *28*, 31–36.

[2] M. Moret, L. Friedrich, F. Grisoni, D. Merk, G. Schneider, *Nat. Mach. Intell.* **2020**, *2*, 171–180.

[3] H. M. Berman, T. Battistuz, T. N. Bhat, W. F. Bluhm, P. E. Bourne, K. Burkhardt, Z. Feng, G. L. Gilliland, L. Iype, S. Jain, P. Fagan, J. Marvin, D. Padilla, V. Ravichandran, B. Schneider, N. Thanki, H. Weissig, J. D. Westbrook, C. Zardecki, *Acta Crystallogr. Sect. D Biol. Crystallogr.* **2002**, *58*, 899–907.

[4] L. A. Solt, T. P. Burris, *Trends Endocrinol. Metab.* **2012**, *23*, 619–627.

[5] S. Hochreiter, J. Schmidhuber, *Neural Comput.* **1997**, *9*, 1735–1780.

[6] D. P. Kingma, J. L. Ba, in *3rd Int. Conf. Learn. Represent. ICLR 2015 - Conf. Track Proc.*, International Conference On Learning Representations, ICLR, **2015**.

[7] U. S. The MathWorks Inc., MATLAB 2017a, Natick, Massachusetts, **2017**.

[8] F. Grisoni, G. Schneider, *Methods Mol. Biol.* **2021**, *2266*, 11–35.

[9] H. L. Morgan, *J. Chem. Doc.* **1965**, *5*, 107–113.

[10] F. Pedregosa, G. Varoquaux, A. Gramfort, V. Michel, B. Thirion, O. Grisel, M. Blondel, P. Prettenhofer, R. Weiss, V. Dubourg, J. Vanderplas, A. Passos, D. Cournapeau, M. Brucher, M. Perrot, É. Duchesnay, *J. Mach. Learn. Res.* **2011**, *12*, 2825–2830.

[11] T. Rodrigues, D. Reker, P. Schneider, G. Schneider, *Nat. Chem.* **2016**, *8*, 531–41.

[12] M. Feher, J. M. Schmidt, *J. Chem. Inf. Comput. Sci.* **2003**, *43*, 218–227.

[13] J. Heering, D. Merk, *Methods Mol. Biol.* **2019**, *1966*, 175–192.

[14] S. Willems, W. Kilu, X. Ni, A. Chaikuad, S. Knapp, J. Heering, D. Merk, *Commun. Chem.* **2020**, *3*, 85.
